# Hybrid NIR-responsive liposome/hydrogel platform mediating chemo-photothermal therapy of retinoblastoma enhanced by quercetin as an adjuvant

**DOI:** 10.7150/thno.108471

**Published:** 2025-03-10

**Authors:** Min Lin, Xiumei Liu, Jing Li, Hong Zou, Jie Wang, Zi Yan, Ying Liu, Yaqi Lyu, Nianping Feng

**Affiliations:** 1 School of Pharmacy, Shanghai University of Traditional Chinese Medicine, Shanghai, 201203, China.; 2 Department of Ophthalmology, Shuguang Hospital Affiliated to Shanghai University of Traditional Chinese Medicine, Shanghai, 201203, China.

**Keywords:** Retinoblastoma, chemo-photothermal therapy, hydrogel, intravitreal injection, NIR-controlled release

## Abstract

**Rationale:** Retinoblastoma is one of the most aggressive paediatric cancers originating from retina and finally invades vitreous humour or other tissues if treated improperly. Current interventions often fail due to the complexities of tumour progression and drug resistance driven by frequent intravitreal injection and epithelial-mesenchymal transition (EMT). Consequently, there is an unmet need for more effective, less invasive treatments.

**Methods:** A near-infrared (NIR)-responsive liposome/hydrogel platform that incorporates quercetin (QUE) and doxorubicin (DOX) co-loaded liposomes (QD Lipo), and indocyanine green (ICG) in a low-gelling temperature agarose hydrogel (LAgel) was developed to improve efficacy through localized, on-demand delivery of chemo-photothermal therapy directly at the site of tumour. The initial phase of the study examined the injectability, reversibility and stability of QD Lipo/ICG/LAgel under NIR using *cryo*-SEM technique and rheological measurement. Following this, photothermal conversion capability and controlled release of QD Lipo, alongside the mobility and penetration of QD Lipo was investigated using infrared thermal imaging, nanoparticle tracking analysis and fluorescence microscope. Moreover, retinoblastoma orthotopic model was established to corroborate the anti-tumour effectiveness *in vivo*. Flow cytometry, H&E, immunohistochemical staining, animal imaging and western blotting were performed to identify the underling mechanism of QD Lipo/ICG/LAgel to improve the treatment.

**Results:** The thermosensitivity and photothermal conversion capabilities of QD Lipo/ICG/LAgel enable precise, on-demand drug delivery in vitreous, significantly reducing the need for frequent intravitreal injections. *In vitro* and *in vivo* evaluation demonstrated that with facilitation of QUE, our platform effectively targets the rapid tumour progression and overcome therapeutic resistance of mild photothermal therapy (PTT), by modulating EMT process and inhibiting heat shock protein (HSP) level.

**Conclusions:** This innovative approach not only mitigates the current challenges of repeated invasive medication, but also sets new strategies for treating complex ocular diseases.

## Introduction

Retinoblastoma is a rare, aggressive cancer that predominantly affects the retina of young children, typically under the age of five. The global incidence of retinoblastoma ranges between 1/15,000 to 1/20,000, with significant disparities in mortality rates depending on the economic development level of the region where the patient resides. In low-income countries, the mortality rate among children with retinoblastoma was around 60% whereas in upper-middle-income countries, the mortality rate is less than 20% 1, 2. Over the years, the management of retinoblastoma has significantly advanced with strategies including systemic chemotherapy, as well as intra-arterial and intravitreal delivery of chemotherapeutics to minimize systemic side effects. In advanced cases, treatments may extend to external beam radiation therapy, cryotherapy, and thermotherapy. Despite these advancements, challenges persist in cases presenting with vitreous seeds or extraocular spread, where the cancer invades beyond the retina into the vitreous humour, optic nerve, brain, and distant organs, or exhibits resistance to standard therapies 2, 3.

Increasing evidence shows that epithelial-mesenchymal transition (EMT) played a vital role in tumour metastasis. In retinoblastoma, EMT reduces cell-cell adhesion, enabling tumour cells to detach from the retina and seed in the vitreous cavity. This seeding is problematic as it widely disperses the tumour cells, making localized treatments less effective and increasing the risk of recurrence. Moreover, cells undergoing EMT also acquire resistance to therapeutic interventions, complicating treatment at advanced stages 4-6. Therefore, it is highly desired to develop effective therapies that can simultaneously address those multiple risk factors such as rapid tumour growth, metastasis and drug resistance.

Quercetin (QUE), a flavonoid present in various fruits, vegetables, and other plant-derived substances, is known to process numerous beneficial effects such as antioxidant, anti-inflammatory, immunomodulatory, vascular protection, neuroprotection and anticancer properties 7. These characteristics have led to its extensive application in the treatment of various ocular diseases, including allergic conjunctivitis, cataracts, glaucoma, and retinal disorders, either as a standalone treatment or in combination with other therapeutics 8. However, the potential benefits of QUE as an adjuvant to therapies such as chemotherapy or thermotherapy for retinoblastoma have not yet been demonstrated, probably due to the lack of effective carriers for simultaneously delivering QUE and other therapeutics. Accordingly, an ocular delivery platform to carry multiple therapeutics is in dire need for multi-treatments of retinoblastoma.

Intravitreal injection of chemotherapeutics is considered the most effective method for treating retinoblastoma with vitreous seeds, as it bypasses blood-retinal barriers and ensures localized, high concentration at the lesion. However, frequent injections are required due to rapid vitreous clearance, posing risks such as infection, bleeding, retinal detachment, even secondary tumour metastasis 9, 10. To overcome these challenges, injectable materials for sustained dosing, such as ocular implants or temporary drug reservoirs have been widely studied and developed in recent years 11. Hydrogels, derived from natural polymers like hyaluronic acid, alginate and chitosan, have shown excellent biocompatibility, biodegradability, and mechanical properties. Their ability to mimic the vitreous matrix and the porous structure make them effective platforms to work as drug depot for sustained delivery in vitreous 12, 13. However, it is challengeable to load hydrophobic drug such as QUE directly into the hydrogel. Liposomes are nanomedicines that have been proved highly successful with great biosafety and biodegradability. They can carry both hydrophilic and lipophilic drugs simultaneously in a single vesicle. Liposomes have been proven to have excellent vitreous penetration and prolonged retention time in recent studies 14-16. Accordingly, loading small molecular drug in liposome/hydrogel hybrid system is believed to be an effective way to overcome both frequent injection and rapid clearance after release from hydrogel.

Drug delivery systems activated to release drugs at target sites using external stimuli hold great promise for improving the treatment of many diseases. External stimuli can be repeatedly switched on and off at desired time to release drug to match patient's needs. Light, in particular, with its non-invasive nature and high spatial and temporal control, has been explored in ocular applications 17-19. Indocyanine green (ICG) is a fluorescent dye that frequently used in ophthalmology for evaluation of retinal disorders and tumours 20. ICG subjects to good photo-thermal conversion when absorbing the near-infrared of 808 nm. Thus it has wide applications with thermosensitive materials and in the field of photothermal therapy (PTT) 21.

**Figure 1:**
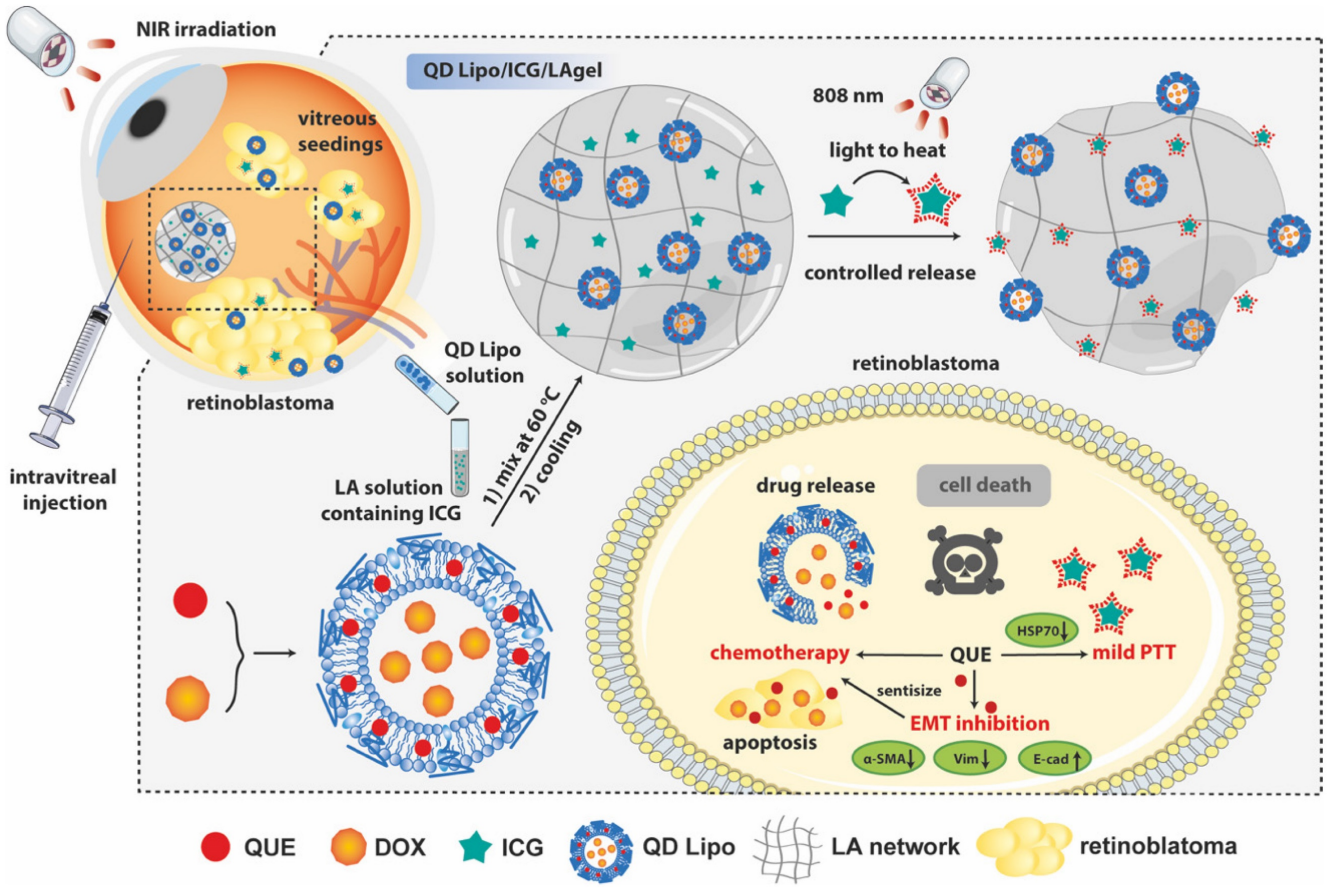
Schematic illustration of the formation of QD Lipo/ICG/LAgel and the mechanism of NIR-triggered synergistic chemo-photothermal therapy of retinoblastoma adjuvated by QUE after intravitreal administration.

In our study, an NIR-responsive hybrid liposome/hydrogel platform made from low-gelling temperature agarose (LA) encapsulating QUE and DOX co-loaded liposomes (QD Lipo) and ICG was developed for synergistic chemo-photothermal therapy of retinoblastoma (Figure 1). LA hydrogel (LAgel) is a biocompatible and biodegradable gel derived from agar, a natural polymer extracted from seaweed. LAgel undergoes reversible sol-gel transitions based on temperature changes 22, 23. Under NIR irradiation, the encapsulated ICG converts light energy to heat, thus enabling the disassembly of LAgel, leading to the controlled release of QD Lipo for chemotherapy. Simultaneously, mild PTT treatment could also be achieved considering the heat generation, if the temperature is exquisitely tuned. Unlike traditional intraocular hydrogel with single function, QD Lipo/ICG/LAgel not only works as a drug reservoir after intravitreal injection, but also accomplishes simultaneous chemo-photothermal therapy through remote light control. Compared to the existing retinoblastoma therapy requiring repeated injections, our multifunctional ocular delivery platform reduces the vitreous invasiveness to a minimal level and enhances therapeutic outcomes by achieving multiple effects concurrently.

## Methods

### Materials and reagents

Hydrogenated soy phosphatidylcholine (HSPC) and 1,2-distearoyl-sn-glycero-3-phosphoethanolamine-N-[methoxy(polyethylene glycol)-2000] (DSPE-mPEG2000) were purchased from Shanghai Advanced Vehicle Technology Co., Ltd (Shanghai, China). Cholesterol (Chol), doxorubicin (DOX), agar, low gelling temperature agarose (LA) were purchased from Aladdin (Shanghai, China). Quercetin (QUE, > 95% purity) was acquired from Sigma-Aldrich (St. Louis, MO, USA). Indocyanine green (ICG) was provided by Bidepharm (Beijing, China). Hyaluronic acid (HA, 200-400 kDa was purchased from Bloomage (Beijing, China). Coumarin-6 (C6) and haematoxylin&eosin were acquired from Sigma-Aldrich (St. Louis, MO, USA). Fetal Bovine Serum (FBS) was obtained from FuHeng Biology Biotechnology Co., Ltd (Shanghai, China). RPMI 1640, Dulbecco's modified Eagle's medium/F12 (DMEM/F-12), 100× penicillin-streptomycin (P/S) antibiotics, 4,6-diamino-2-phenyl indole (DAPI), Cell Counting Kit-8** (**CCK-8), BCA protein assay kit, 5× loading buffer, 10% SDS-PAGE gel, rainbow protein marker, tris buffered saline (TBS), ECL solution and D-fluorescein potassium salt were purchased from Meilunbio (Liaoning, China). Annexin V-FITC apoptosis kit, 7-aminoactinomycin D (7-AAD), RIPA lysis buffer and skimmed milk powder were provided by Beyotime, (Shanghai, China). Anti-vimentin (Vim) primary antibody and anti-α-smooth muscle actin (α-SMA) primary antibody were purchased from Cell Signalling Technology (Boston, USA). anti-hear shock protein (HSP)70 primary antibody was purchased from Proteintech (Wuhan, China). Anti-Ki67 primary antibody was acquired from Abcam (Cambridge, UK). Anti-GAPDH primary antibody and goat anti-rabbit lgG (H+L) HRP conjugated secondary antibody were purchased from Origene (Rockville, USA). Gentamicin sulphate eye drop was obtained from Dirui Pharmaceutical Co., Ltd (Jilin, China). 4% paraformaldehyde was obtained from Keygen (Jiangsu, China). Ethanol, methanol, ammonium sulphate, phosphotungstic acid and sodium chloride were provided by Sinopharm (Shanghai, China).

### Cells and animals

Human retinoblastoma cells (Y79) and Y79-GFP-luc were purchased from FuHeng Biology Biotechnology Co., Ltd (Shanghai, China). Y79 and Y79-GFP-luc were cultured in RPMI-1640 supplemented with 20% FBS and 100 units/mL P/S antibiotics at 37 °C with 5% CO_2_ supply_._ Human retinal pigment epithelial cells (ARPE-19) were purchased from MeilunBio (Liaoning, China). ARPE-19 were cultured in DMEM/F-12 supplemented with 10% FBS and 100 units/mL P/S antibiotics at 37 °C with 5% CO_2_ supply.

BALB/c nude mice (female, 16-20 g) and SD rats (male, 200-220 g) were obtained from the Experimental Animal Centre of Shanghai University of Traditional Chinese Medicine and fed in SPF condition with food and water. All the animal experiments were performed in accordance with the protocol approved by the institutional animal care and use committee of Shanghai University of Traditional Chinese Medicine (PZSHUTCM2309180003, PZSHUTCM2309070004).

### Preparation of QD Lipo and other labelled liposomes

QD Lipo was prepared using thin film hydration method followed by probe sonication. In detail, HSPC, Chol, DSPE-mPEG2000 and QUE were dissolved in anhydrous ethanol at room temperature, the molar ratio of HSPC, Chol and DSPE-mPEG2000 was 71.25:23.75:5, and the molar ratio of HSPC and QUE was 20:1. Ethanol was evaporated under reduced pressure using a rotary evaporator in a 45 °C water bath to obtain a blank phospholipid film, the phospholipid film was then dried in a 37 °C vacuum oven for over 8 hours. Once dried completely, DOX solution was added to hydrate the phospholipid film at 75 °C for 30 min. Subsequently, the solution was sonicated with the probe for 10 minutes to obtain QD Lipo. Coumarin-6 labelled liposome with DOX (CD Lipo) or without DOX (C6 Lipo) and were prepared by the same procedure.

### Characterization of QD Lipo

After dilution of QD Lipo, the size, polydispersity index (PDI), and zeta potential were measured using a Zetasizer (Nano ZS Series, Malvern Instrument, UK) under conditions of 25 °C. Encapsulation efficiency and drug loading rate were measured by UV spectrum at the absorbance wavelength of 371 nm and 492 nm for QUE and DOX, respectively, followed by calculations according to the reference 24. To test the stability, QD Lipo was placed in water bath at 37 °C, 45 °C and 67 °C, respectively. Samples were withdrawn at 5 min, 15 min, 30 min and 60 min to measure the size and PDI. The morphology of QD Lipo was observed with a TEM (JEM-1400 Plus, Tokyo, Japan). Prior to TEM examination, samples were dropped onto the carbon mesh (Zhongjingkeyi, China) and stained with 2% (v/v) phosphotungstic acid for 1 min. The excess liquid was removed with filter paper and dried at 25 °C. To study the release of QUE and DOX from QD Lipo, 500 μL of QD Lipo were transferred into a dialysis bag (MWCO 8000-14000) and incubated in 5 mL of PBS (pH 7.4) containing 1% Tween 80 at 37 °C. At predetermined intervals, the medium of samples was withdrawn and substituted with the same volume of fresh medium. QUE and DOX were quantified by measuring the absorbance at 371 nm and 392 nm, respectively.

### Movement of liposome in simulated vitreous humour (SVH)

0.25 g of hyaluronic acid and 0.2 g of agar were dissolved in 100 mL ultrapure water with constant stirring for 15 min under 100 °C of water bath. The SVH was obtained after cooling down to room temperature. CD Lipo was injected into SVH and PBS and kept at 37 °C. The particle size distribution and particle displacement were tracked using NanoSight (NS300, Malvern Instrument, UK). The mean square displacement (MSD) values were calculated according to Eq. (1). The MSD of the particle movement over 100 frames was calculated to assess the diffusion capability of the particles.


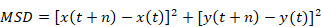

(1)

where “*n*” represents the interval between frames (24.9825 fps), “t” represents a certain time point, and “x” and “y” represent the coordinates of the particle within that frame, respectively.

### C6 Leakage

To investigate the C6 leakage from C6 Lipo, 500 μL of C6 Lipo were placed into a dialysis bag (MWCO 8000-14000) and incubated in 5 mL of PBS (pH 7.4) supplemented with 1% Tween 80 at 37 °C. The medium was withdrawn and replaced with an equivalent volume of fresh medium at certain time point. C6 fluorescence signal was quantified at the *E_X_* wavelength of 492 nm and the *E_M_* wavelength of 515 nm.

### Cellular uptake and cell aggregates penetration

Y79 cells (1×10^5^) were seeded in 12-well plates and grown for 48 h. Then the cells were treated with CD Lipo and other formulations. The concentration of C6 and DOX was 0.5 μg/mL and 5 μg/mL, respectively. After incubation for 1 h and 4 h, the cells were washed with PBS and the fluorescence intensity of C6 and DOX was measured using flow cytometer (CytoFLEX S, Beckman Coulter, USA). Y79 cell aggregates were transferred into a confocal dish and cultured overnight. The cell aggregates were treated with CD Lipo and other formulations for 2 h. Then the medium containing liposomes was removed, and cell aggregates were washed with PBS gently. Subsequently, cells were fixed with 4% paraformaldehyde for 15 minutes, followed by staining with DAPI for 20 min. After washing with PBS for 3 times, the fluorescence signals of C6 and DOX were collected under CLSM (TCS SP8, Leica, Germany) using the Z-tack mode with a step size of 5 μm.

### Preparation of QD Lipo/ICG/LAgel and other hydrogels

ICG was first dissolved in water at the concentration of 1 mg/mL as stock solution. LA was dissolved in water at certain concentrations under 70 °C, then various volumes of ICG stock solution and QD Lipo were added to LA solution, followed by cooling down to room temperature. To optimize the final formulation of QD Lipo/ICG/LAgel, the concentration of LA was prepared in the range from 0.1% to 10% (w/w). The concentration of ICG loaded in QD Lipo/ICG/LAgel were 0.1 mg/mL, 0.2 mg/mL and 0.4 mg/mL, respectively. The ratio between phospholipid in QD Lipo and LAgel was 0.25:1, 0.5:1, 1:1 and 2:1 (w/w). Other hydrogels containing ICG (ICG/LAgel), QD Lipo (QD Lipo/LAgel), CD Lipo (CD Lipo/LAgel) or blank liposome and ICG (Lipo/ICG/LAgel) were prepared using the same method.

### Characterization of QD Lipo/ICG/LAgel

To test the injectability of the hydrogel, certain amount of LA was dissolved in water to obtain the final concentrations of LA solution with 0.1%, 0.5%, 1%, 2%, 3%, 4%, 5% and 10% at 70 °C. ICG was added to LA solution at the concentration of 0.4 mg/mL with constant agitation. Then ICG/LA solution was transferred to the injection syringe. After gelling in the syringe, ICG/LAgels were injected into PBS, photos were taken 5 min after injection.

To determine the phase-transition temperature (T_m_) and heat capacity of LA, DSC scanning was performed using a three-step method (DSC25, TA instrument, USA). First, a crucible with LA power was balanced at -10 °C for 10 min, then heat flow change from -10 °C to 100 °C with stepwise of +5 °C /min was measured and exothermic curves was plotted. Sapphire and empty crucible were measured using the same method as control.

The morphology of QD Lipo/ICG/LAgel was observed using *cryo*-scanning electrical microscope (*cryo*-SEM). QD Lipo/ICG/LAgel was dehydrated by gradient ethanol solutions with concentrations of 30%, 50%, 70%, 90% and 100%, and dried using a critical point dryer (EM CPD300, Leica, Germany). QD Lipo/ICG/LAgel was adhered to conductive carbon tape on sample stage and snap-frozen in liquid nitrogen for 30 min. After sublimating at -90 °C for 10 min, the sample was coated with gold and observed via *cryo*-SEM (Helios G4 UC, Thermo Fisher, USA) under an acceleration voltage of 5 kV. The temperature of the stage was -140 °C.

The storage and loss moduli of QD Lipo/ICG/LAgel were measured using a rheometer (MCR101, Anton Paar, China) equipped with a 50 mm diameter plate geometry. Oscillatory strain sweeps (10 rad/s), oscillatory frequency sweeps (0.1 % strain), time sweeps with high and low temperature cycles (0.1% strain, 10 rad/s) and temperature sweeps from 25 °C to 80 °C (0.1% strain, 10 rad/s) were performed.

To ensure the stability of QD Lipo after release, 2 mL of QD Lipo/LAgel was placed into a glass vial, to which 3 mL of PBS was added. The vial was then left at room temperature. Observations were made over 72 h and photos were taken at certain time. After the final time point, the supernatant was collected to measure the particle size and make TEM observation.

### Photothermal effect of QD Lipo/ICG/LAgel

To ensure the optimal mode of NIR irradiation, ICG/LAgel was prepared with the ICG concentrations of 0.1 mg/mL, 0.2 mg/mL and 0.4 mg/mL, respectively. Then, an 808 nm NIR laser (MDL-H-808, CNIlaser, China) with output powers of 1 W, 2 W, and 3 W was used to irradiate ICG/LAgel from a distance of 45 mm for 60 min. For *in vivo* test, ICG/LAgel was intravitreally injected into the eyes of Balb/c nude mice, followed by irradiation with NIR laser of 808 nm for 20 min. The infrared thermal images and temperature changes of the ICG/LAgel were recorded using an infrared thermographic camera (PI400, Optris Germany). To calculate the photothermal conversion efficiency, 5 cycles of “on-off” NIR irradiation of 808 nm was performed on QD Lipo/ICG/LAgel. Temperature change of the irradiation and cooling process was recorded. Photothermal conversion efficiency after the 1^st^ cycle and the 5^th^ cycle of NIR exposure was measured as described previously according to Eq. (2), Eq. (3) and Eq. (4) 25.


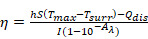

(2)


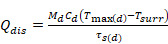

(3)


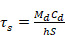

(4)

Where “η” represents photothermal conversion efficiency, “h” is the heat transfer coefficient, “S” is the surface area of the container, “T_surr_” is the ambient temperature of the surroundings, “T_max_” is the maximum steady-state temperature, “I”is the output power of laser, and A_λ_ is the absorbance at the wavelength of λ. “Q_dis_” represents the heat dissipation. M_d_ and C_d_ represent the mass and heat capacity of the dispersing medium, respectively. The heat capacity of LA was 2.84J/(g・K) according to Figure S1 measured by DSC scanning. τ_s_ represents the time constant for heat transfer in the system, which was determined in Figure S2.

### NIR triggered release of QD Lipo/ICG/LAgel

200 μL of C6 Lipo/ICG/LAgel was placed at the bottom of the Eppendorf tube and 2.8 mL of PBS containing 1% Tween-80 was added as the release medium. An 808 nm of NIR laser with a power of 2 W was used for intermittent irradiation (5 min of irradiation followed by 5 min without irradiation). Without irradiation, the samples were kept in the dark. 100 μL of release medium were withdrawn every 5 min to determine the content of C6 in the supernatant, and an equal volume of the release medium was replenished. The cumulative release of C6 at different time points was calculated using the following Eq. (5) and Eq. (6). Subsequently, the cumulative release curve over time was plotted.


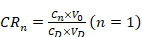

(5)


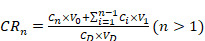

(6)

where “*n*” represents the time point, “C*_n_*” is the content of C6 measured at the *n^th^* time point, “V_0_” is the total volume, “C_D_” is the concentration of C6 in the formulation, “V_D_” is the volume of the formulation, and “V_1_” is the sampling volume.

### Cell viability

Y79 cells were seeded in a 96-well plate at the density of 1×10^4^ cells per well. After 48 h, the cells were treated with drug containing formulations at various concentration. To study the PTT effect, ICG was added to achieve the final ICG concentration of 0.4 mg/mL. The concentration of QUE and DOX for PTT study was 1 μM and 2 μM, respectively. For irradiation groups, the cells were irradiated with an 808 nm NIR laser at a power of 2 W and a distance of 45 mm for 0 min, 3 min, 5 min and 10 min, after which the cells were continued to be cultured for 48 hours. For ARPE-19 cells, a density of 5×10^3^ cells per well was seeded in 96-well plate and incubated for 24 h. LA solution, ICG solution and NIR irradiation was performed to treat the cells. Cell viability of both Y79 and ARPE-19 cells was measured using CCK-8 reagent at the absorbance of 450 nm using a microplate reader (Spectramax iD5, Molecular Devices, USA).

### Y79 apoptosis

Y79 apoptosis was detected by Annexin V-FITC/7-AAD staining kit. In brief, Y79 cells were seeded in a 6-well plate at a density of 2×10^5^ per well and grown for 24 h. Then, the cells were treated with different drug-loaded formulations at a QUE concentration of 1 μM or a DOX concentration of 2 μM. For irradiation groups, the cells were irradiated with an 808 nm NIR laser at a power of 2 W and a distance of 45 mm for 5 min. After 24-h incubation, the cells were washed with cold PBS for 3 times. Prior to flow cytometry analysis, the cells were suspended in 195 μL of binding buffer and then stained with Annexin V-FITC/7-AAD according to the protocol.

### Western blotting

The harvested Y79 cells, or Y79 tumours were incubated in RIPA buffer on ice for 30 min, and centrifuged at 16000 g for 30 min. The concentration of total protein was measured by a BCA protein assay kit. Then the proteins adjusted to the same concentration by RIPA buffer dilution in each sample was mixed with loading buffer and boiled for protein denaturation. The sample proteins were separated on 10% sodium dodecyl sulphate polyacrylamide gel electrophoresis (SDS-PAGE), and then transferred to a nitrocellulose membrane. After rinsed with TBS-T solution, the membrane was incubated in blocking solution containing 5% skim milk powder at for 1 h. Subsequently, the membrane was incubated with primary antibodies of anti-Vimentin (1:1000 dilution), α-SMA (1:1000 dilution), E-cadherin (1:1000 dilution), HSP70 (1:5000 dilution) or GAPDH (1:5000 dilution) at 4 °C overnight and a secondary antibody at r.t. for 2 h. Finally, immunoreactive proteins were visualized, and images were acquired with an Imaging System (Touch Imager, E-BLOT, China) after staining with ECL kit. Integrated optical density was calculated using the Fiji analysis software (Image J 1.54f, Wayne Rasband and contributors, National Institutes of Health, USA).

### Intravitreal injection

Rats or mice were anesthetized using an inhalation anaesthesia machine (R500, RWD Life Science, China) with isoflurane. Post-anaesthesia, animals were placed on a heating pad and the eyes were wet around with sterilized PBS. QD Lipo/ICG/LAgel or other formulations were injected into the posterior eye using an insulin syringe with a 30-gauge needle. During injection, the needle was perpendicular to the eye surface and the injection depth was kept around 2-3 mm. When the intravitreal injection was finished, the needle was slowly removed from the eyeball and gentamicin eye drops were administered to prevent infection.

### *In vivo* distribution and release

SD rats were anesthetized and intravitreally injected with 10 μL of CD Lipo/ICG/LAgel. CD Lipo and saline were injected as control. For irradiation group, the rats were irradiated with 808 nm NIR laser from a 45 mm distance for 5 min with a power of 2 W daily after the injection. At 10 min and 48 h post-injection, the rats from each group were euthanized, and eyeballs were enucleated to make the frozen section with a thickness of 20 μm. After sectioning, the slides were fixed with 4% paraformaldehyde for 10 min, stained with a DAPI solution for 15 min, and rinsed with TBS. The fluorescence of eyeball sections was observed under a cell imaging system (BioTek Cytation 5, Agilent, USA).

### Anti-tumour effects in Y79 orthotopic model

2 μL of a Y79-GFP-Luc cell suspension was injected into the eyes of Balb/c nude mice following the intravitreal injection procedure. The intraocular bioluminescence was measured to monitor the tumour growth. Tumour-bearing mice were intraperitoneally injected with D-luciferin potassium salt solution at the dose of 150 mg/kg, and then anesthetized with isoflurane. After 20 min, the bioluminescence signal was detected using IVIS imaging system (IVIS spectrum, Perkin Elmer, USA). According to the intraocular bioluminescence, tumour-bearing mice were divided into 6 groups (n = 6) and treated with 2 μL of different preparations via intravitreal injection. NIR irradiation was performed on Day 0, 2, 4, 6 and 8 with a power of 2 W and a distance of 45 mm for 5 min. During the treatment period, the bioluminescence was detected using the same method and the body weight of mice was recorded. At the end of treatment (on Day 17), the mice were sacrificed to collect eye tissues for further experiments. For histological analysis, the tumours were cut into 5 μm thick sections and treated with haematoxylin and eosin (H&E), and the sections were observed under microscope (KF-PRO-120, KFBio, China). Cell proliferation was detected using immunohistochemical staining using Ki67 antibody. The isolated tumour was fixed in 4% paraformaldehyde and embedded in paraffin to prepare sections of 5 μm thickness. Then, an immunohistochemistry test was performed according to the standard instructions. The sections were observed and quantified under microscope in three representative fields. IHC Profiler software was used to analyse the positive cell ratios.

### Safety evaluation

Blank liposome and Lipo/ICG/LAgel were injected into the eye of rats. Rats injected with saline were used as control. NIR irradiation without injection was also performed to treat the eye. Additionally, rats injected in the eye with Lipo/ICG/LAgel were subjected to daily exposure to NIR laser with a power of 2 W and a distance of 45 mm for 5 min. The rats were euthanized on Day 0, 1, 3 and 7 post-injection and the eyeballs were collected. Paraffin sections of collected eyes were prepared and H&E staining was carried out according to the standard protocol 26.

### Statistical Analysis

Data are presented as the means ± SD. Differences between the groups were assessed by one-way ANOVA and Independent-samples t-test using GraphPad Prism 10. The criterion for statistical significance was taken as ^*^p < 0.05, ^**^p < 0.01 and ^***^p < 0.001.

## Results and Discussion

### Characterization and photothermal response of ICG/LAgel

**Figure 2:**
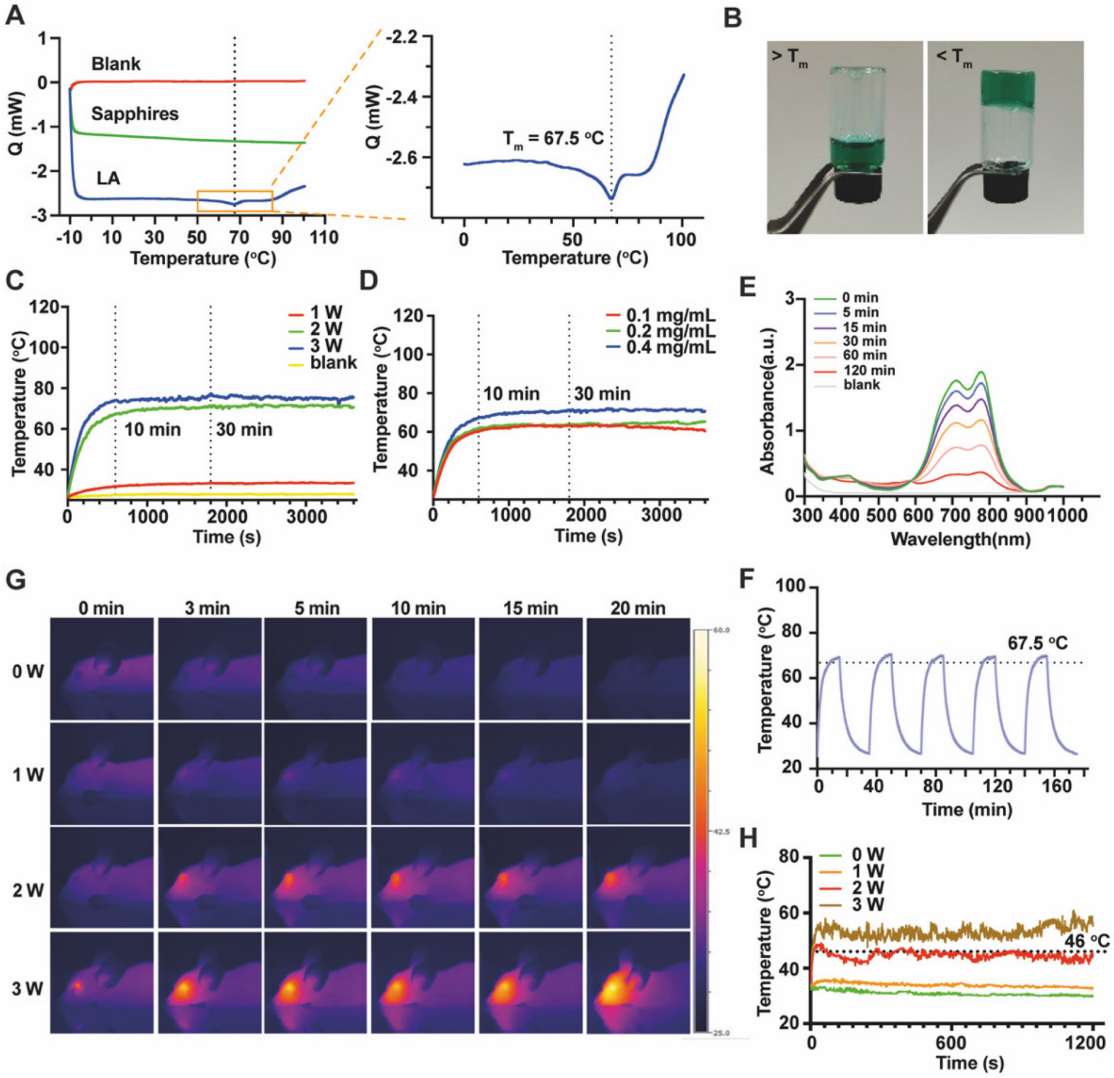
Characterization and NIR responsiveness of ICG/LAgel. (A) Images of DSC scanning of LA. (B) Appearance of ICG/LAgel at the temperature above T_m_ or below T_m_. (C) Temperature change under NIR irradiation with output power of 1 W, 2 W and 3 W. (D) Temperature change of ICG/LAgel under NIR irradiation at ICG concentration of 0.1 mg/mL, 0.2 mg/mL and 0.4 mg/mL. (E) UV absorbance of ICG solution under NIR irradiation within 120 min. (F) Temperature change under “on-off” NIR irradiation cycles. (G) Thermal photos of the mice's eyes after intravitreal injection of ICG/LAgel under NIR irradiation. (H) Quantitative temperature change of the mice's eyes in Figure 2G. In NIR irradiation, the distance between laser and sample was 45 mm.

To obtain the LAgel with injectability, a series of concentrations of LAgel was prepared for injection. 0.4 mg/mL ICG was loaded in LAgel to facilitate observation. LAgel with concentration of 0.1% (w/w) cannot formed the hydrogel at room temperature. However, when the concentration of LAgel is higher than 5%, the LA solution became highly viscous even at 70 °C (Figure S3). A syringe equipped with 30G needle was used to test the injectability since 30G is the needle size clinically used for intravitreal injection 27. To simulate *in vivo* vitreous component, simulated vitreous humour (SVH) consists of 0.25% hyaluronic acid and 0.2% agar was prepared according to a previous report 28. LAgel lost the injectability when the concentration is higher than 5%, while LAgel with concentrations of 0.5%-2% cannot form compact hydrogel in SVH after injection (Figure S4). Considering the ease of injection, 3% LAgel was finally chosen to prepare QD Lipo/ICG/LAgel. From DSC measurement, the endothermic peak appeared at 67.5 °C, showing T_m_ of LA (Figure 2A). Above T_m_, ICG/LAgel presented in liquid form while below T_m_, green ICG/LAgel was formed at the bottom of a glass bottle, demonstrated by the inversion test (Figure 2B).

Then, we investigated the ICG concentration and NIR intensity to find optimal photothermal response. The distance between NIR source and samples was fixed at 45 mm. Thermal images showed ICG/LAgel exhibited significant photothermal response similar to that of ICG solution (Figure S5). Upon irradiation with NIR of 808 nm, the temperature of ICG/LAgel increased rapidly, reaching a peak within 10 min and the peak temperature was maintained for over 30 min as irradiation continues. However, the irradiation with output power of 1 W was insufficient to induce an adequate photothermal response with the peak temperature less than 34 °C (Figure 2C). At 2 W, ICG/LAgel with ICG concentration of 0.4 mg/mL reached a higher peak temperature, approximately 5 °C higher than 0.1 mg/mL and 0.2 mg/mL (Figure 2D). During NIR irradiation, the absorption of ICG at wavelength of 695 nm and 800 nm under UV-Vis spectrum gradually decreased, indicating that ICG was depleted under NIR exposure, and this depletion was time-dependent (Figure 2E). Accordingly, a cyclic “on-off” NIR irradiation was performed and photothermal conversion efficiency after 5 cycles of NIR exposure was investigated. According to the obtained temperature change (Figure 2F) and Eq.(2)-(4), the photothermal conversion efficiency of ICG/LAgel after the 1^st^ cycle and the 5^th^ cycle of NIR irradiation was determined to be 14.58%, and 12.25% respectively. Nevertheless, this reduction had neglectable influence on reaching T_m_ of 67 °C in each cycle (Figure 2F). ICG is known for its poor water solubility, susceptibility to photobleaching, and rapid clearance from the body, typically within 2-4 min of circulation 29. When ICG absorbs NIR light, it undergoes excitation and can interact with oxygen molecules in the environment to produce highly reactive ROS, leading to the breakdown of ICG molecule structure. Additionally, ICG's tendency to aggregate, especially in high concentrations, can further accelerate this breakdown 30, 31. In our study, encapsulating ICG within the LAgel matrix helped keep ICG in a dispersed state, preventing its aggregation and reduce the ROS formation. Moreover, LAgel encapsulation can protect ICG from direct interaction with light, further minimizing the photobleaching process, enhancing the stability of ICG.

Considering the safety of heat generation in vitreous, we further verified the photothermal response of ICG/LAgel *in vivo*. 5 μL of ICG/LAgel was intravitreally injected into healthy nude mice followed by NIR treatment. The peak intraocular temperature was achieved within 1 min and it is dependent on the power of NIR laser (Figure 2G-H). 1 W of NIR irradiation resulted in a minimum thermal effect with temperatures only 3 to 5 °C higher than the non-irradiated group. 2 W of NIR stably produced a temperature around 46 °C, which is the appropriate temperature allowing for mild PTT. In contrast, NIR irradiation at 2 W without ICG/LAgel injection resulted in a temperature of around 37.5 °C, close to the normal physiological temperature. However, exposure to 3 W of NIR irradiation resulted in intraocular temperatures exceeding 43 °C, which may cause potential tissue damage (Figure S6A & S6B). To balance ICG consumption and maintain an effective and safe PTT temperature, 0.4 mg/mL ICG was finally loaded in ICG/LAgel and NIR irradiation with output power of 2 W was chosen for therapy. Previous studies have reported that the maximum permissible power density of an 808 nm laser for ocular tissue is 0.3 W/cm² 32. Accordingly, the NIR intensity was measured under the selected experimental conditions. At a distance of 45 mm between the NIR laser and the samples, the NIR intensity was found to be 0.28 W/cm², which is below the safety threshold, confirming that the NIR irradiation in our settings is safe for ocular tissue.

### Preparation and characterization of QD Lipo

**Figure 3:**
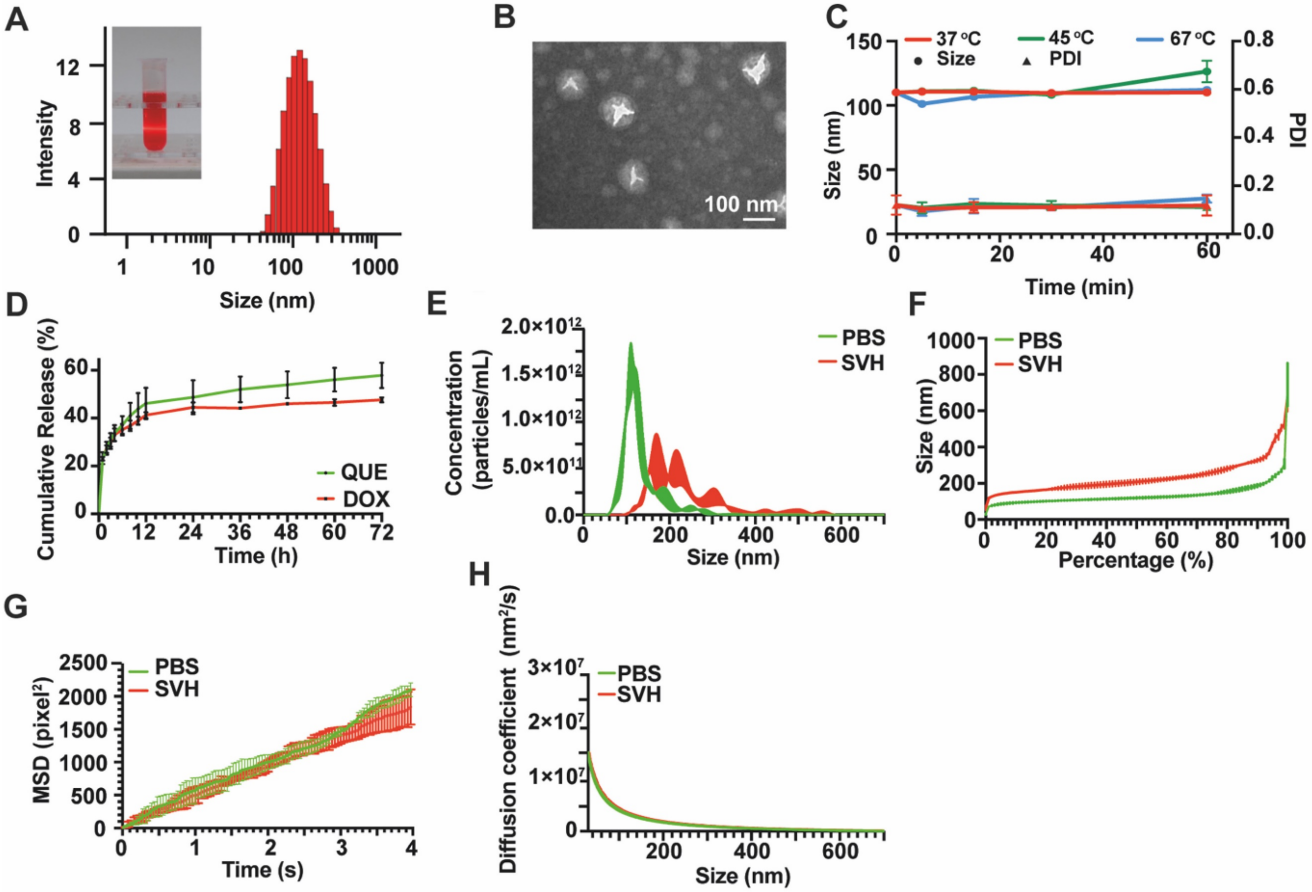
Characterization of QD Lipo. (A) Size distribution and appearance of QD Lipo. (B) TEM image of QD Lipo. (C) Size and PDI change of QD Lipo at the temperature of 37 °C, 45 °C and 67 °C. (D) *In vitro* release profiles of QUE and DOX from QD Lipo. (E) Size distribution of CD Lipo in SVH and PBS. (F) Percentage distribution of different size of CD Lipo. (G) Brownian motion trajectories of CD Lipo in SVH and PBS within 4 s. (H) Mean square displacement (MSD) of CD Lipo in SVH and PBS calculated from Figure 3G.

To achieve the co-delivery of QUE and DOX and prolong their retention in the vitreous, QD Lipo was prepared according to our previous study. After purification by ultrafiltration, UV spectrum revealed that the encapsulation efficiency of DOX and QUE was 91.17±4.41% and 96.20±4.40%, respectively. The drug loading rates were 2.19±0.11% for DOX and 1.26±0.06% for QUE. QD Lipo had a diameter of 107.87±0.13 nm with a polydispersity index (PDI) of 0.13±0.02 (Figure 3A). A spherical morphology with bilayer structures of QD Lipo was confirmed by TEM analysis, and the diameter was approximately 100 nm (Figure 3B), being consistent with the previous DLS result. The surface charge of QD Lipo was determined as -36.53±0.61. To assess the stability, QD Lipo was exposed to temperatures of 37 °C, 45 °C, and 67 °C, corresponding to physiological temperature, mild PTT temperature, and the preparation temperature of QD Lipo/ICG/LAgel, respectively. After 1 hour, the increase in particle size was minimal, with an observed change of approximately 10 nm (Figure 3C). These findings indicated that QD Lipo maintains its integrity within these temperature ranges. QUE and DOX release behaviour were studied at 37 °C to mimic the temperature of ocular tissue. During the release period, both QUE and DOX showed a sustained release pattern from QD Lipo, at a release rate of 57.88% and 47.61%, respectively (Figure 3D).

Vitreous is a gel-like substance supported by long fine collagen fibres, proteins and polysaccharides. The movement of particles in vitreous humour might be hindered due to the crosslinking vitreous network 33, 34. Hence, SVH was used to assess the mobility of our liposome. To ensure an accurate detection, C6 fluorescence probe, and DOX co-labelled liposome (CD Lipo) was injected in SVH with particle density of around 10^13^/mL. Compared to PBS, the particle size of CD Lipo increased to an average of 229±7.21 nm in SVH, with 46% of nanoparticles were under the size of 200 nm and nearly 92% of nanoparticles were under 350 nm (Figure 3E-F). CD Lipos with small size and large size were selected to record their Brownian motion with in 4 s. Representative mobility tracks were presented in Figure 3G. The distribution of track length showed no significant difference between small sized CD Lipo in SHV (SHV-s) and in PBS (PBS-s) with respective mean sizes of 131.6 nm and 135.4 nm. Trajectories of large sized CD lipo with mean size of 292.3 nm span shorter distances within the time scale of the measurement. Further analysis was assessed through Mean Square Displacement (MSD), whereas a greater MSD value suggests a larger positional change within the unit time, indicating stronger mobility. The MSD value of small sized CD Lipo within 4 s was 1838.84±266.86 pixel^2^, with no marked difference observed compared to 2099.51±104.28 pixel^2^ in PBS. In contrast, large sized CD Lipo displayed reduced MSD with 1230.77±61.76 pixel^2^, which were 1 time slower than the small sized CD Lipo in SHV, indicating that the SHV poses a minor barrier for the liposomes (Figure 3H). Previous study has shown that large particles (2 μm) were found to remain in vitreous cavity after intravitreal injection, but smaller particles (< 400 nm) move without restraint 35. Vitreous is a weak barrier for the diffusion of negatively charged lipid-based formulation 36. Given that our liposome is anionic and nearly all have a diameter of less than 400 nm, it is believed that after released from hydrogel, QD Lipo has the potential to move freely in the vitreous and reach the tumour site.

### Cellular uptake and penetration

**Figure 4:**
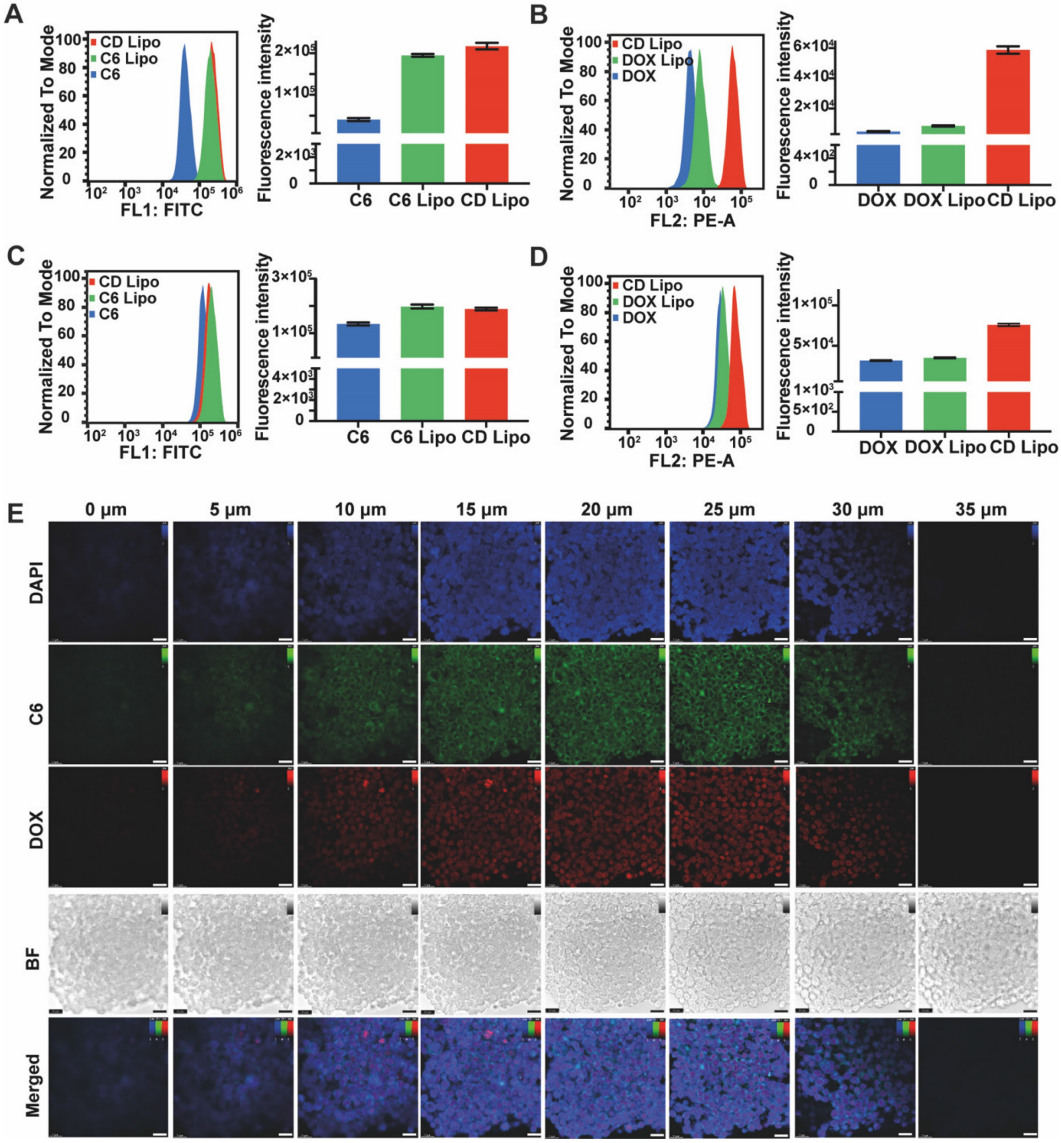
Cellular uptake and penetration of QD Lipo. (A-D) Time-dependent cellular uptake of CD Lipo in Y79 measured by flow cytometry. C6 fluorescence at 1 h (A), DOX fluorescence at 1 h (B), C6 fluorescence at 4 h (C), DOX fluorescence at 4 h (D). (E) Confocal images of Y79 aggregates after 2-h incubation with CD Lipo. The scale bar is 20 μm. (Green: C6 signal, Red: DOX signal, Blue: DAPI signal from staining of cell nuclear)

After demonstrating the mobility of QD Lipo in the vitreous, it is crucial to determine whether QD Lipo would enter the tumour in order to deliver therapeutic effects. Y79 cells, a cell line derived from human retinoblastoma tumour, was chosen as *in vitro* model. First, cellular uptake was measured using flow cytometry. After incubation for 1 h, the fluorescence intensity of C6 Lipo treated cells and DOX Lipo treated cells increased by 4.77-fold and 1.80-fold, respectively, compared to free C6 and DOX, showing significant enhancement in uptake when in liposome (Figure 4A-B). After 4-h incubation, the fluorescence intensity of all treated cells increased, indicating a time-dependent uptake (Figure 4C-D). In addition, the dual-loaded CD Lipo demonstrated a more significant fluorescence compared to DOX and DOX Lipo (Figure 4B, 4D), we hypothesized that with addition of C6 loaded in the phospholipid layer, the alteration in the fluidity of liposome membrane has resulted in the increased capability of cellular uptake.

In retinoblastoma, tumour cells exhibit cluster growth characteristics. During vitreous seeding, tumour cells leave retina and disperse to form aggregates in vitreous cavity. Thus, the therapeutic efficacy of anti-tumour drug is contingent upon their successful delivery to the interior regions of tumour. To study the penetration of QD Lipo, Y79 cell aggregates were collected and incubated with CD Lipo for 2 h. Z-tack scanning by CLSM revealed both green and red fluorescence distributed uniformly throughout Y79 aggregates, indicating the deep penetration and co-delivery of CD Lipo (Figure 4E).

### Preparation and characterization of QD Lipo/ICG/LAgel

QD Lipo/ICG/LAgel was prepared by directly mixing QD Lipo and ICG in melted LA solution, followed by cooling down to r.t. for LA gelation. To verify the stability of QD Lipo/ICG/LAgel, various amount of QD Lipo was loaded in LAgel and left to stand for 8 days. Distinct stratification was observed in QD Lipo after intense centrifugation, resulting in the destruction of QD Lipo. However, QD Lipo /LAgel maintained good homogeneity without stratification after centrifuge regardless the ratio between QD Lipo and LA, suggesting enhanced stability compared to free QD Lipo (Figure S7). Considering the increased viscosity of QD Lipo solution with higher concentrations which would lead to uneven dispersion of QD Lipo in LAgel. Thus, a ratio between phospholipid in QD Lipo and LAgel at 1:1 (w/w) was chosen for QD Lipo/ICG/LAgel.

**Figure 5:**
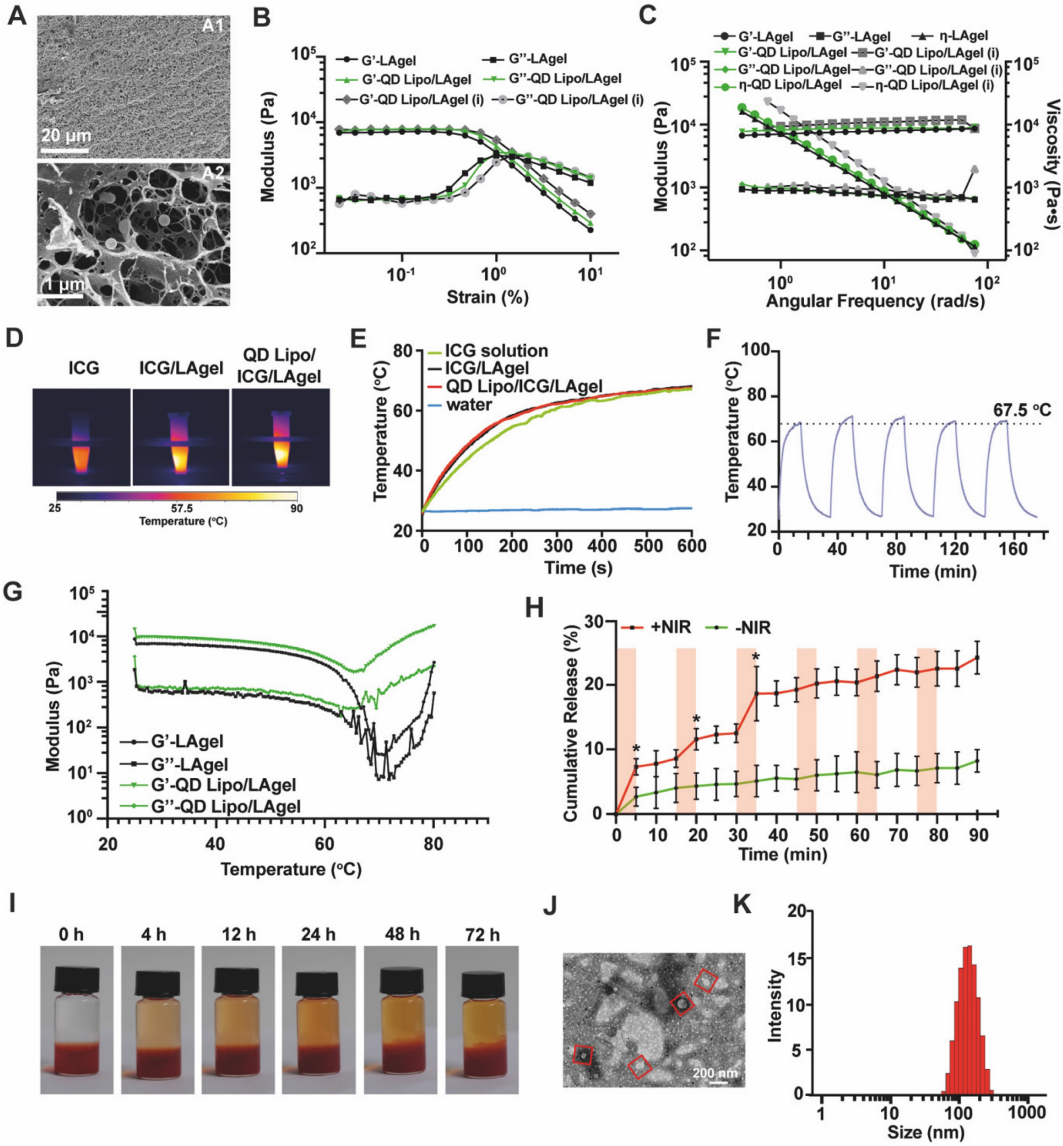
Characterization of QD Lipo/ICG/LAgel. (A) *cryo-*SEM image of QD Lipo/ICG/LAgel. (B) Strain sweep of LAgel, QD Lipo/LAgel and QD Lipo/LAgel after injection at fixed frequency of 10 rad/s. (C) Frequency sweep of LAgel, QD Lipo/LAgel and QD Lipo/LAgel after injection at fixed strain of 0.1%. (D) Thermal images of ICG solution, ICG/LAgel and QD Lipo/ICG/LAgel under NIR irradiation of 808 nm for 10 min. The concentration of ICG was 0.4 mg/mL. (E) Temperature change of ICG solution, ICG/LAgel and QD Lipo/ICG/LAgel within 10 min recorded from Figure 5D. Water was used as blank control. (F) Temperature change under “on-off” NIR irradiation cycles. (G) Temperature sweep of LAgel or QD Lipo/LAgel at fixed strain of 0.1% and fixed frequency of 10 rad/s. (H) Cumulative release of C6 Lipo/ICG/LAgel under periodic NIR irradiation of 808 nm. Light red colour indicates the NIR was in the mode of “on”. (I) Diffusion of QD Lipo over time from QD Lipo/LAgel. (J) TEM image and (K) size distribution of the collected media in diffusion study of Figure 5I after 72 h. Red squares indicate liposomes in TEM image.

A porous morphology of QD Lipo/ICG/LAgel was confirmed by *cryo*-SEM. Compared to the SEM images of bare LAgel (Figure S8), several spherical structures were found in the space of the hydrogel porous network, showing the distribution of QD Lipo in LAgel (Figure 5A). Oscillatory rheology was applied to assess the hydrogel formation and whether liposome loading would affect the mechanical property of LAgel. Strain sweeps demonstrated the formation of hydrogel since the storage modulus (G') is higher that loss modulus (G'') when below the critical strain point. While when the strain exceeded this point, a pronounced decrease in G' signifies the collapse of the hydrogel structure (Figure 5B). In frequency sweep, both G' and G'' remained nearly unchanged over a range of frequencies. These results implied the viscoelastic behaviour of LAgel and the hydrogel integrity is maintained across those frequencies, which may resulted from the highly cross-linked network of LAgel (Figure 5C).

Moreover, QD Lipo/LAgel exhibited similar G' and G'', indicating that the incorporation of liposomes into the LAgel did not affect its mechanical properties (Figure 5B-C). To further evaluate the hydrogel condition after injection, QD Lipo/LAgel was loaded onto the rheometer plate via syringe injection, followed by rheological assessment. Both strain sweeps at a fixed frequency (10 rad/s) and angular frequency sweeps at a fixed strain (0.1%) demonstrated that G' and G'' remained nearly unchanged compared to the QD Lipo/LAgel prior to injection, suggesting a rapid gel recovery after injection process (Figure 5B-C).

ICG is widely used photosensitive molecular that coverts light to heat upon NIR irradiation. When irradiated with NIR of 808 nm, QD Lipo/ICG/LAgel was observed to heat up rapidly, and the temperature reached 45 °C within 2 min. Moreover, QD Lipo/ICG/LAgel possesses the same efficient photothermal response capability as ICG solution and LAgel (Figure 5D-E). Under cyclic on-off irradiation, QD Lipo/ICG/LAgel presented unattenuated photothermal responsiveness during the 5 repeated irradiation, reaching the temperature of 67 ^o^C, indicating QD Lipo in hydrogel had no effect on the transition temperature (Figure 5F, 2F). To understand the state of LAgel under phase transition, a temperature sweep in rheological characterization with fixed stain and frequency was measured. The modulus of LAgel and QD Lipo/LAgel decreased gradually with the temperature being raised, indicating that the hydrogel network began to loosen with increasing temperature. When the temperature is over 65 ^o^C, G'of both LAgel and QD Lipo/LAgel decreased sharply and became lower than G'', suggesting a phase transition occurred, which is consistent with the result of DSC (Figure 5G, 2A).

To better understand whether QD Lipo/ICG/LAgel undergoes changes after several rounds of NIR irradiation, the morphology after irradiations was observed under *cryo*-SEM and the image was shown in Figure S9A. Compared to the morphology before irradiation in Figure 5A, porous network was maintained after NIR irradiation. Very few spherical structures were found in the porous network, indicating the structure of hydrogel was reversible and most of the QD Lipo have been released during irradiation process. Additionally, the rheological properties of irradiated QD Lipo/ICG/LAgel were also measured. The nearly unchanged modulus further confirmed that QD Lipo/ICG/LAgel did not undergo significant changes after irradiation, indicating the reversibility of hydrogel network (Figure S9B).

### Release of QD Lipo

To verify the controlled release of encapsulated liposome upon NIR irradiation, fluorescence labelled C6 Lipo (C6 Lipo) was used to prepare the hybrid liposome/hydrogel. C6 showed a leakage of less than 5% from C6 Lipo, thereby ensuring that C6 predominantly remained within the liposomes (Figure S10). This minimal leakage allowed for precise tracking of liposome in the following release study, as the detected fluorescence primarily originated from the C6 Lipo. Then, the release profile of C6 Lipo under NIR irradiation was carried out by immersing C6 Lipo/ICG/LAgel in PBS (pH 7.4) containing 1% Tween 80. After NIR irradiation, C6 Lipo/ICG/LAgel released its payload in a faster pattern than that without irradiation, and the release rate slowed down to normal level when the irradiation was off, which might be resulted from the reformation of LAgel. A 2.84-fold increase in cumulative release was observed after 6 times of cyclic irradiation, compared to the natural release. The distinct step-like variations of cumulative release curves indicated NIR-controlled release of C6 Lipo/ICG/LAgel (Figure 5H).

As the result of release profile showing slow release in normal condition, a visualized release without NIR irradiation was performed using QD Lipo/LAgel. The removal of ICG was to better observe the colour change in the medium caused by QD Lipo. Slight diffusion of red-coloured QD Lipo was observed in the above medium, which gradually increased over time (Figure 5I). Previous study reported that liposomes, with their membrane flexibility, could be released naturally from various hydrogel networks without affecting the integrity of the hydrogel network. And this process is influenced by the density of the hydrogel network, and the composition of the liposomes 37. In QD Lipo/ICG/LAgel, the size of QD Lipo is observed less than the mesh size of LAgel (Figure 5A), leading to slow diffusion of QD Lipo from LAgel in natural condition. To validate the integrity of QD Lipo after release, TEM was applied to observe the morphology. Spherical structure with an approximate diameter of 124.7 nm were detected in the release medium, which matched the sizes of QD Lipo embedded in LAgel (Figure 5J-K). Therefore, it can be considered that in addition to the photothermal effect induced by ICG, QD Lipo/LAgel itself serves as an excellent sustained release system, capable of slowly releasing liposomal drugs over an extended period.

### *In vitro* cytotoxicity and synergistic therapeutic effect

**Figure 6:**
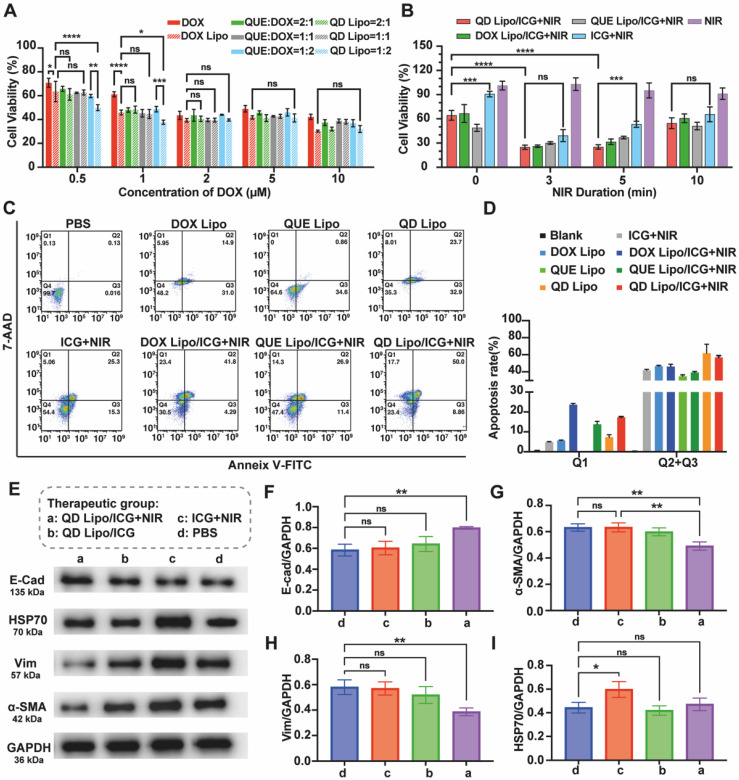
*In vitro* antitumor efficacy of QD Lipo/LAgel. (A) Y79 cell viability was assessed by CCK-8 assay with treatment of free DOX, DOX Lipo, free DOX and QUE combination and QD Lipo at different concentrations for 48 h. The molar ratio between QUE and DOX was 2:1, 1:1 and 1:2 (means ± SD, n = 3). (B) Y79 cell viability after ICG, DOX Lipo/ICG, QUE Lipo/ICG and QD Lipo/ICG treatment combined with NIR irradiation (means ± SD, n = 3). (C) Apoptosis rate of Y79 cells was determined by Annexin V-FITC/7-AAD staining. The lower-left, lower-right, upper-right, and upper left quadrants represented the viable, early apoptotic, late apoptotic and dead cells, respectively. The incubation was performed at IC_50_ of DOX and the molar ratio between QUE and DOX was 1:2. Y79 cells were treated with different formulations for 48 h. Duration of NIR irradiation was 5 min. (D) Quantification of the dead cells (Q1) and total apoptotic cells (Q2+Q3) in Figure 6C (means ± SD, n = 3). (E) Western blot analysis of E-cad, HSP70, α-SMA and Vim protein expression of Y79 cell lysates after treatment of PBS, ICG+NIR, QD Lipo/ICG and QD Lipo/ICG+NIR for 48 h. GADPH was used as a loading control. (F-I) Quantitative analysis of the expression level of the protein in Figure 6E (means ± SD, n = 3). E-cad (F), α-SMA (G), Vim (H) and HSP70 (I). The group label a-d is shown in Figure 6E. (^*^P < 0.05, ^**^P < 0.01, ^***^P< 0.001, ^****^P< 0.0001)

To understand the anti-tumour effect adjuvated by QUE in the system, cell viability in the presence of NIR irradiation or not was first examined using CCK-8 assay. First, QUE and DOX with various molar ratios (2:1, 1:1 and 1:2) were encapsulated in QD Lipo and then incubated with Y79 cells. Combination of free QUE and DOX, free DOX alone and DOX Lipo were used as different controls. Shown in results (Figure 6A), DOX Lipo exhibited more profound cytotoxicity than DOX, mainly due to the improved uptake of liposome. Compare the cell viability between different ratios of QUE and DOX treated cells, the most effective combination of QUE and DOX was 1:2, especially in the form of liposome. Thus, QUE and DOX with molar ratio of 1:2 was used for the following studies. Surprisingly, this inhibition of cell viability was observed when the concentration of DOX is below 2μM. The increased cytotoxicity of QUE and DOX at relatively low concentration has also been reported for treating melanoma 38, indicating the adjuvant effect of QUE to DOX in killing cancer cells. Given that the limited volume of vitreous cavity restricts administration dosage, therefore the ability of drug to exert potent anti-tumour effects at low concentrations is beneficial for intraocular application. Subsequently, the effect of formulations combined with NIR irradiation was tested. NIR irradiation presented to have no influence on Y79, with cell viability higher than 90%. However, when NIR irradiation was performed together with ICG, Y79 cell viability decreased significantly, showing the photothermal effect induced by ICG was able to inhibit tumour growth. However, prolong the NIR irradiation time could not improve the killing effect on Y79 cells, thus we chose 5 min as NIR duration (Figure 6B).

To analyse the apoptosis of Y79, quantitative measurement by flow cytometry showed that the apoptotic rate of QD Lipo treated Y79 was 61.8%, with an increase of 15.0% compared to DOX Lipo treatment, which implies the addition of QUE in liposome improved the sensitivity of DOX to Y79 (Figure 6C-D). The intervention of PTT had no significant impact on the apoptotic effect induced by QD Lipo but made big differences in cell phenotyping. Upon the introduction of NIR, a substantial clustering of cells was observed in the Q1 region, indicative of necrosis. One of the mechanisms reported from previous reports showed that heat generated by the PTT of photosensitizers can destroy the organelles of tumour cells, resulting in ablation of the tumour and leading to cell necrosis 39.

Mortality in retinoblastoma patients mainly resulted from the metastasis of cancer cells, in which EMT plays the vital role. E-cadherin (E-cad) is a protein that mediates the adhesion between cells through calcium-dependent interactions. In the process of tumour metastasis, decrease in E-cad can reduce cell-cell adhesion, and lead to the detachment of cells from primary tumour. α-SMA (a marker of myofibroblast-like phenotype) and Vimentin (Vim, an intermediate filament protein typically expressed in mesenchymal cells) are mesenchymal-like markers that are usually overexpressed in EMT-activated cells 40, 41. Here, the effect of different formulations on EMT in terms of α-SMA, Vim as well as E-cad was investigated. Western blot assay demonstrated that sole PTT treatment had no effect on inhibiting EMT process (Figure 6E). QD Lipo combined with PTT increased the expression of E-cad (Figure 6F) while α-SMA and Vim was significantly decreased (Figure 6G-H), showing the capability of QD Lipo to reverse EMT process. Further analysis by treating Y79 cells with single drug loaded liposome or with combination of PTT was preformed (Figure S11A). QUE Lipo significantly reduced α-SMA and Vim expression and increased E-cad expression, regardless of its combination with PTT. The downregulation of α-SMA and upregulation of E-cad was also observed in DOX Lipo treated Y79 cell, however, it is less profound compared to QUE Lipo treatment and QD Lipo treatment (Figure S11B-D). These results revealed that QUE played a pivotal role in reversing the EMT process in Y79 tumour cells. This is accomplished by decreasing mesenchymal-like cell characteristics and upregulating the levels of E-cadherin protein, thereby enhancing cell-cell adhesion and potentially reducing the risk of tumour metastasis.

HSP70 refers to a family of proteins known as Heat Shock Proteins of approximately 70 kDa in size. In organisms exposed to fluctuating or high temperatures, the expression of HSP70 is often upregulated as a protective response. This enhanced expression is not only a marker of heat stress but also a crucial adaptive mechanism that contributes to the survival of tumour cells under such conditions 42, 43. WB demonstrated that PTT increased the expression level of HSP70. However, the addition of QUE Lipo and QD Lipo decreased the expression of HSP70 to normal level where as QD Lipo treated cells showed the lowest expression of HSP70 (Figure 6I, S11E). These finding demonstrated that QD Lipo is capable of reducing HSP levels in retinoblastoma, thereby enhancing the sensitivity of tumour cells to temperature fluctuations. This reduction in lethal temperature threshold facilitates the targeted killing of tumour cells via mild PTT without affecting normal tissues 44.

### *In vivo* drug release and distribution

**Figure 7:**
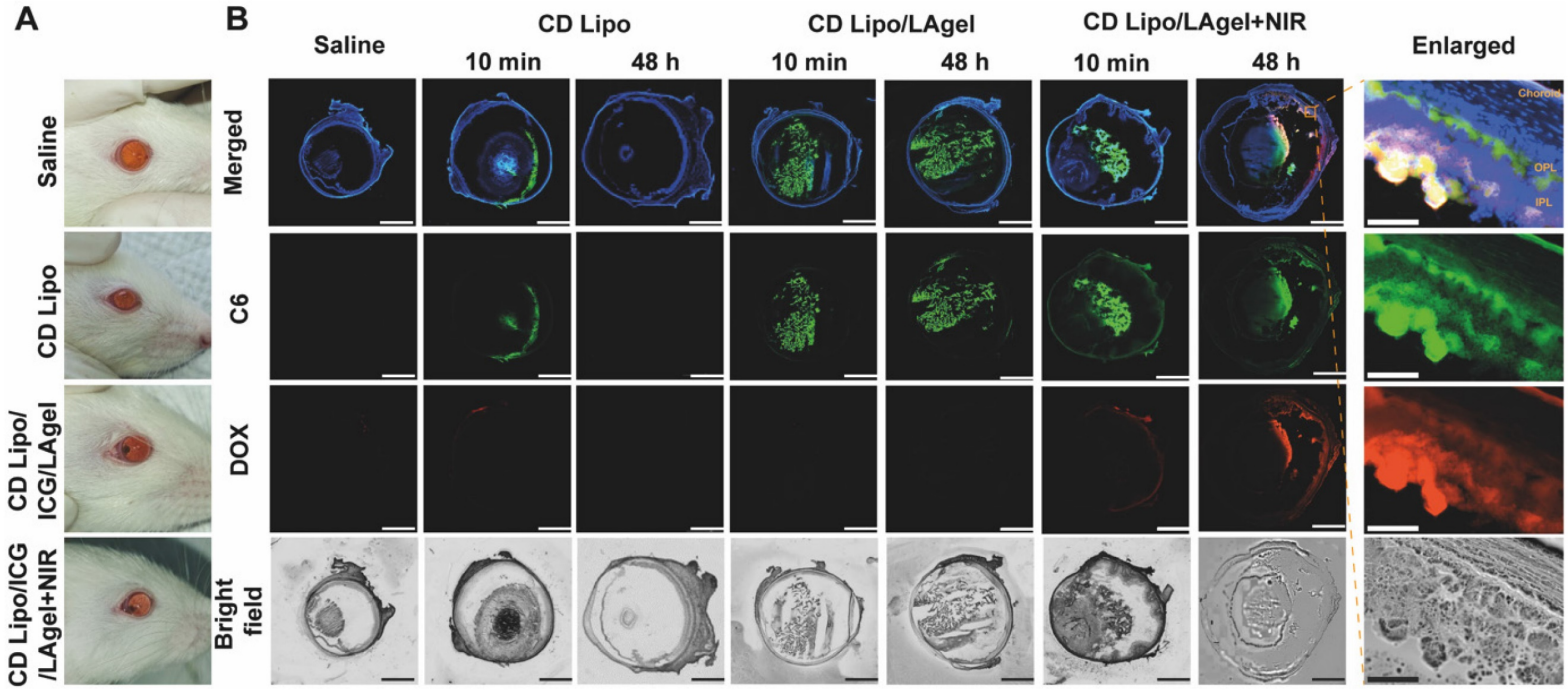
*In vivo* drug release and distribution. (A) Photos of rat's eyes after intravitreal injection of saline, CD Lipo and CD Lipo/ICG/LAgel. The injection volume of the hydrogel was 2 μL. (B) Fluorescence distribution of C6 and DOX after intravitreal injection and NIR irradiation. Eyeball frozen sections were obtained from isolated eye tissues harvested from rats 10 min or 48 h after injection. The scale bars are 2 mm and 100 μm (enlarged), respectively. (Green: C6 signal, Red: DOX signal, Blue: DAPI signal from staining of cell nuclear).

Before verification of the anti-tumour effect of QD Lipo/ICG/LAgel *in vivo*, NIR-controlled drug release and subsequent vitreous distribution was first assessed. Fluorescence labelled liposome (CD Lipo) and hybrid liposome/hydrogel (CD Lipo/ICG/LAgel) were intravitreally injected to the rat's eyes (Figure 7A). Fluorescence signals of both C6 (green) and DOX (red) were visible immediately post-injection of CD Lipo, but disappeared after 48 h, indicating that CD Lipo were likely cleared from the eye (Figure 7B). For CD Lipo/ICG/LAgel, C6 fluorescence could be found post-injection and remained detectable after 48 h in the vitreous. However, C6 fluorescence in the non-irradiated group was more localized with less diffusion compared to the group with NIR irradiation. Additionally, DOX fluorescence was almost undetectable without irradiation, suggesting that DOX might not have been effectively released from the hydrogel. In contrast, C6 and DOX diffused extensively in the eye after 10-min irradiation, and the fluorescence signal of both C6 and DOX remained detectable after 48 h, reaching the areas of inner plexiform layer (IPL) and outer plexiform layer (OPL) of the retina and choroid (Figure 7B). These findings implied that although liposomes can quickly distribute throughout the vitreous due to their small size, they are subject to rapid clearance, which could potentially limit their clinical application for long-term treatment. The most important finding of the current results is that prolonged drug levels were achieved in the vitreous and the retinal tissues when the liposome/hydrogel hybrid platform formed. The LAgel matrix offers a protective shield for the entrapped liposomes, impeding their breakdown and preventing their leakage into vitreous. Upon remote NIR application, the increased temperature from photothermal conversion of ICG loosen the hydrogel structure and improve the mobility of QD Lipo, leading to rapid release and accumulation of therapeutic liposomes in the vitreous. This controlled release behaviour, along with its non-invasive nature, highlights its promising potential for clinical application.

### *In vivo* antitumor efficacy

**Figure 8:**
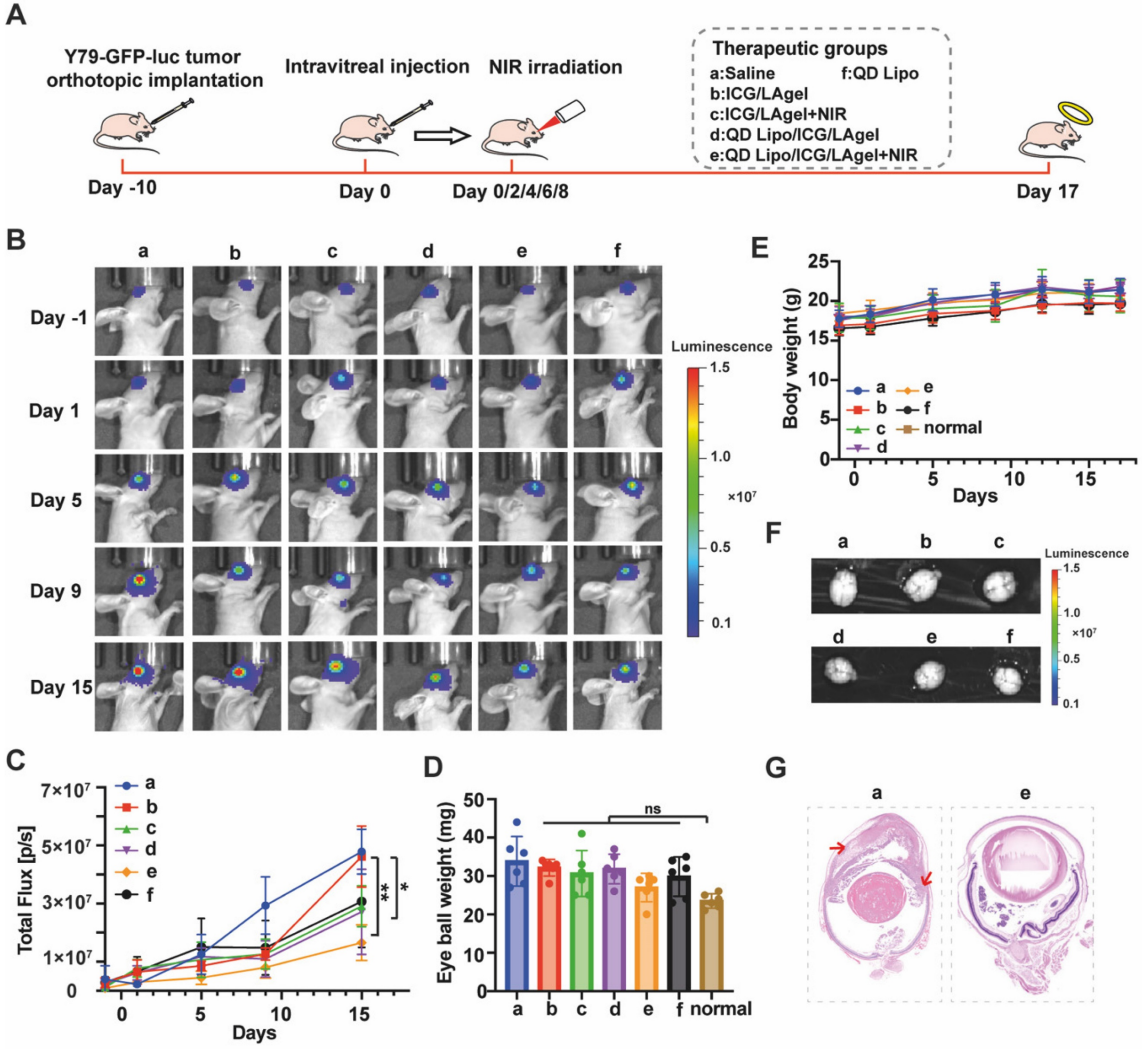
*In vivo* anti-tumour efficacy. (A) Illustration of the animal administration regimen. NIR irradiation was performed after formulation treatment for 5 min. The output power of NIR irradiation was 2 W and the irradiation distance was 45 mm. (B) Representative *in vivo* bioluminescence images of the eyes in orthotopic Y79-GFP-luc tumour-bearing BALB/c nude mice on day -1, 1, 5, 9, and 15. (C) Tumour growth curve presented by the intensity of bioluminescence in Figure 8B. A comparison of the tumour bioluminescence intensity was performed on Day 15 (means ± SD, n = 6). (D) Eyeball weight of eye tissues collected from Y79-GFP-luc tumour-bearing mice on day 17 (means ± SD, n = 6). (E) Body weight changes of Y79-GFP-luc tumour-bearing mice (means ± SD, n = 6). (F) *Ex vivo* bioluminescence images of the brain collected of Y79-GFP-luc tumour-bearing mice on day 17. (G) Representative H&E staining of the harvested eyes in group a and e. Red arrows indicate the metastatic tumour cells. The group label a-f is shown in Figure 8A. (^*^P < 0.05, ^**^P < 0.01, ^***^P< 0.001, ^****^P< 0.0001)

We evaluated the* in vivo* therapeutic efficacy of QD Lipo/ICG/LAgel using an orthotopic retinoblastoma model. Y79 cells transfected with green fluorescent protein and luciferase genes (Y79-GFP-luc) were intraocularly implanted in nude mice. The tumour growth was monitored by the luminescence signals trigged by D-luciferin using IVIS. 10 days after implantation, the Y79-GFP-luc retinoblastoma-bearing mice were randomly divided into 6 groups and intravitreally injected with saline, QD Lipo, ICG/LAgel, ICG/LAgel+NIR, QD Lipo/ICG/LAgel, QD Lipo/ICG/LAgel+NIR, respectively. 808 nm of NIR irradiation was performed every 2 days for 5 times (2 W, 45 mm distance, 5 min) with the first day of NIR irradiation recorded as Day 0 (Figure 8A). During treatment period, the tumour size was measured according to the intensity of luminescence signals (Figure 8B). From tumour growth curves obtained, mice treated with QD Lipo/ICG/LAgel+NIR presented a much slower growth compared to other preparations. On Day 15, the luminescence intensity in the eyes treated with QD Lipo/ICG/LAgel+NIR was significantly lower than those in the groups of saline (3-fold), QD Lipo/ICG/LAgel (1.6-fold) and ICG/LAgel+NIR (1.8-fold), showing the synergistic effect of chemotherapy and photothermal therapy mediated by QD Lipo/ICG/LAgel (Figure 8C). These findings were also reflected by examining the weight of isolated eyeballs on the last day of treatment (Figure 8D). The body weights of the mice were unaltered after treatment, indicating locally delivered formulations did not exhibit significant systemic toxicity (Figure 8E).

As retinoblastoma progresses, it can spread to the vitreous body forming vitreous seeding, and subsequently invade the anterior chamber, the choroid and further metastasizes to brain through optic nerve 6. Therefore, brains and eyeballs were collected and subsequentially analysed for anti-metastasis examination. Live imaging of the nude mice's brains showed no luminescence signals from tumour cells in all groups (Figure 8F), suggesting that brain metastasis had not yet occurred within our modelling period. However, tumour invasion to anterior chamber was found serious in the Model group from H&E staining, whereas in the group treated with QD Lipo/ICG/LAgel+NIR and QD Lipo/ICG/LAgel, tumours were confined to the vitreous and did not invade other tissues (Figure 8G, S12). The invasion in the QD Lipo group was not well controlled, possibly due to the rapid clearance of QD Lipo after intravitreal injection (Figure S12). These results demonstrated that QD Lipo in hydrogel plays a significant role in combating tumour metastasis. In addition, the fluorescence of ICG in mice treated with ICG containing group was detected on the last day of treatment period (Figure S13). The weakened fluorescence showed ICG was consumed under NIR irradiation. Moreover, ICG was still present in the eye demonstrated the ICG in the hydrogel was sufficient to cover the treatment period.

**Figure 9:**
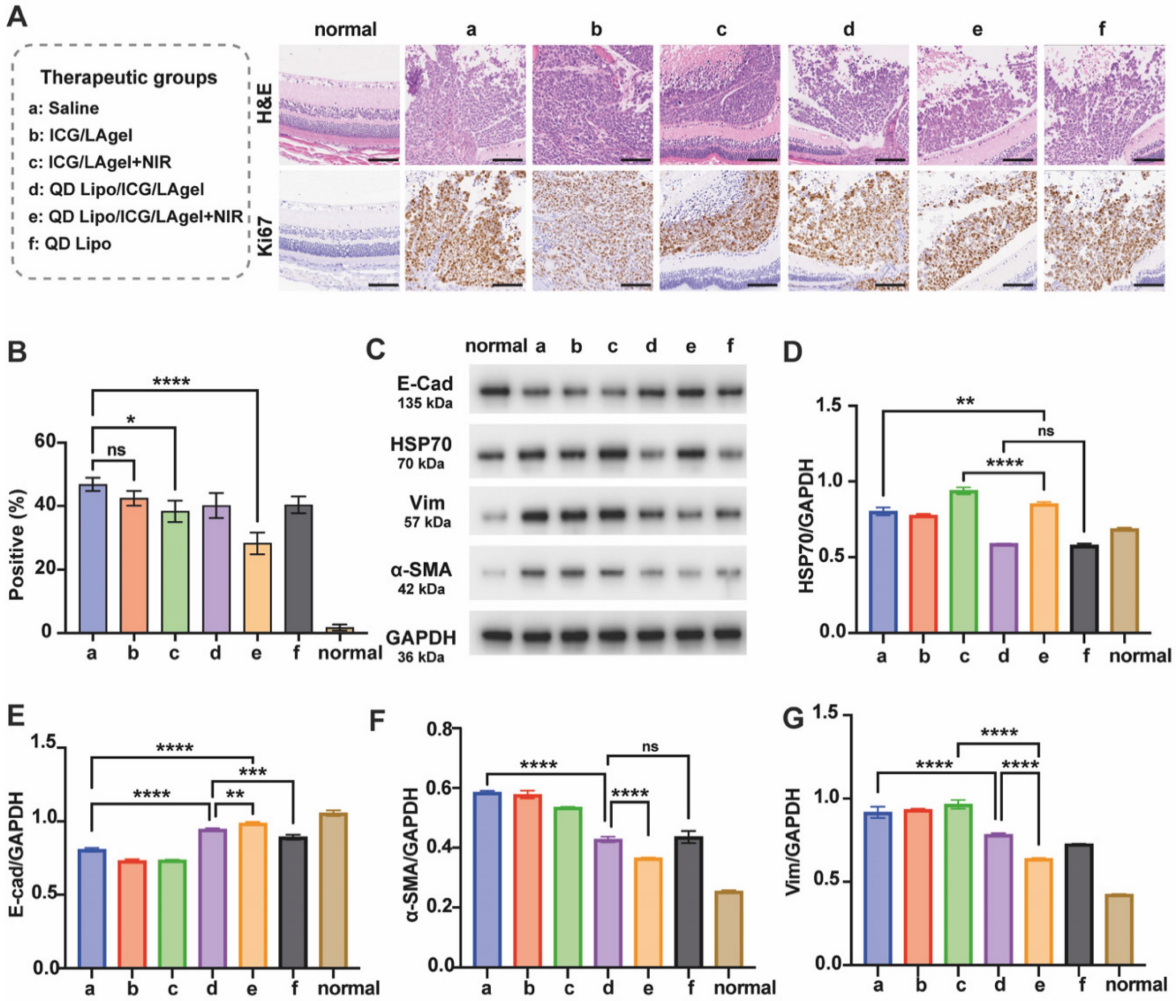
Inhibition of tumour proliferation and EMT *in vivo*. Eyes for these examinations were harvested from retinoblastoma bearing mice on day 17 after administration. (A) H&E staining and Ki67 immunochemical staining of the tumours. Ki67-positive cells are stained brown. The scale bar is 100 μm. (B) Quantitative analysis of Ki67 positive cells in Figure 9A. The quantification was performed by quantifying 3 representative fields of Ki67-immunostained sections under an optical microscope (means ± SD, n = 3). (C) Western blotting of E-cad, HSP70, Vim and α-SMA proteins in tumour tissue lysates. GADPH was used as a loading control. (D-G) Quantification of the blotting bands in Figure 8C (means ± SD, n = 3). HSP70 (D), E-cad (E), α-SMA (F), Vim (G). The group label a-f is shown in Figure 9A. (^*^P < 0.05, ^**^P < 0.01, ^***^P< 0.001, ^****^P< 0.0001)

Paraffin sections of isolated tumours were subjected to H&E staining and immunohistochemical Ki67 assay. QD Lipo/ICG/LAgel+NIR showed the highest percentage of necrosis in H&E staining. The immunohistological staining detected by Ki67 further confirmed the results (Figure 9A). QD Lipo/ICG/LAgel+NIR exhibited a 39.72% reduction in Ki67 positive cells compared to the saline group, showing the most inhibition of tumour cell proliferation (Figure 9B). All these results together demonstrated the potential of QD Lipo/ICG/LAgel+NIR in treating retinoblastoma *in vivo*. The adjuvant effect of QUE was further analysed by WB to explore the mechanism underlying the synergistic anti-tumour efficacy of QD Lipo/ICG/LAgel+NIR (Figure 9C). After NIR irradiation to ICG/LAgel treated eyes, a significant increase in HSP70 expression was observed, indicating that tumour cells were making adaptive changes to resist the increased temperature. However, the addition of QD Lipo suppressed the expression level of HSP70. QD Lipo/ICG/LAgel+NIR treatment significantly downregulated the HSP70 level by 22.15% compared to the ICG/LAgel+NIR group (Figure 9D). The results suggested QD Lipo can suppress HSP70 expression, thereby enhancing the sensitivity of retinoblastoma to mild PTT.

Retinoblastoma exhibited reduced expression of E-cad and increased expression level of Vim and α-SMA, indicating the activation of the EMT pathway (Figure 9E-G). QD Lipo/ICG/LAgel group showed a significant upregulation of E-cad, with an even more pronounced effect when combined with PTT. QD Lipo treatment could also increase the expression level of E-cad, but the upregulation was found not significant in *in vivo* experiments (Figure 9E). For Vim and α-SMA expression, all the preparations containing QD Lipo decreased the expression level, demonstrated significant inhibitory effects on EMT pathway. The suppression was most notable in the QD Lipo/ICG/LAgel+NIR group, with Vim and α-SMA expressions reduced by 35.68% and 39.88%, respectively, compared to the saline group (Figure 9F-G).

Overall, these results demonstrated the capability of QD Lipo/ICG/LAgel+NIR to enhance the therapeutic efficacy of combined chemotherapy and mild PTT in retinoblastoma. The intravitreally injectable delivery platform exploited the photothermal conversion of ICG, enabling the NIR-controlled release of QD Lipo, thereby conferring prolonged and on-demand chemotherapy to the tumour. Meanwhile, this photothermal conversion generates heat, inducing hyperthermia in tumour tissues, leading to cellular damage and tumour reduction. The localized and simultaneous combination treatment maximizes the therapeutic effect. Metastasis is recognized as a contributor to cancer-related mortalities in which EMT has been reported as the initial step for cancer cells to acquire mobility 45. With encapsulation of QUE in liposome, the controlled release also contributes to the accumulation of QUE in tumour cells, leading to EMT inhibition by improving cell-cell adhesion and reducing mesenchymal phenotype, showing the potential of our platform in metastasis inhibition. The therapeutic efficacy was also improved by the function of QUE to support mild PTT. HSP70 is often overexpressed to protect cancer cells from heat induced damage 43, 46. With QUE encapsulated in the liposome/hydrogel platform, it limited tumour development when exposed to NIR by decreasing the HSP70 level in the tumour, thereby providing considerable PTT. Hence, with QUE as an adjuvant, QD Lipo/ICG/LAgel synergistically combated retinoblastoma *in vivo* compared to chemotherapy or PTT alone. Its non-invasive approach further underscores its potential for human retinoblastoma therapy.

### Safety evaluation

The safety of the vehicles and irradiation conditions was studied both *in vitro* and *in vivo*. ARPE-19 is a kind of epithelial cells that forms part of the blood-retinal barrier (BRB) in the eye. Cell viability in the presence of ICG and drug-free LAgel, was approximately 85%-95%, even at high temperature. Moreover, NIR of 808 nm without photothermal transition also presented no harm on ARPE-19 cells, with the average cell viability more than 80% after 5-min irradiation (Figure S14).

Healthy rats were intravitreally injected with drug free preparations and irradiated with NIR. H&E section revealed no pathological changes in rat retina. The photoreceptor and rod cells were arranged neatly with no changes in density or morphology compared to control group injected with saline (Figure S15A). After injection of Lipo/ICG/LAgel followed by NIR irradiation, slight localized microvascular dilation and mild inflammatory cell infiltration were observed on the 1^st^ day of photothermal therapy. However, these phenomena subsided gradually in the following several days (Figure S15B). According to the International Commission on Non-Ionizing Radiation Protection (ICNIRP) guidelines, a temperature of at least 45°C is required to produce a thermal burn on retina. The threshold for lenticular changes caused by IR-A (780 nm-1400 nm) is approximately 50 MJ/m² 47. Another recent study found that NIR light did not cause any significant changes in axial length, suggesting it has no impact on vision 48. Taken together, these findings suggest that NIR irradiation with the current parameters does not adversely affect ocular structures or function.

## Conclusions

In summary, a hybrid NIR-responsive liposome/hydrogel platform for enhanced chemo-photothermal therapy of retinoblastoma was developed. QD Lipo/ICG/LAgel worked as a therapeutic reservoir with close distance to the tumour site after intravitreal injection, avoiding long circulation and penetration of drugs to reach the lesion. Upon remote NIR irradiation, the encapsulated ICG converted light to heat thus loosen the structure of QD Lipo/ICG/LAgel, enabling controlled release of QD Lipo in the vitreous. Subsequently, nanosized QD Lipo diffused freely in the vitreous humour and enhanced the chemotherapeutic effect through directly inducing tumour apoptosis of DOX and inhibiting EMT-process of QUE. Additionally, the inhibition of EMT may also be effective in reducing vitreous seeding and tumour metastasis. At the same time, the increased temperature improved the mild photothermal effect by inhibiting the expression of HSP70 through QUE. The work of QD Lipo/ICG/LAgel provides avenues for preparing less invasive multifunctional intraocular delivery platform and a treatment strategy that simultaneously enhance synergistic chemo-photothermal therapy. However, the *in vivo* degradability and long-term safety of QD Lipo/ICG/LAgel requires further refinement. In the future, the mechanism of action of QD Lipo/ICG/LAgel will be thoroughly investigated, especially on chemotherapy-resistant model. By tailoring the synergistic effects of the payloads, efforts will be made to identify additional applications of this formulation in other ocular diseases.

## Supplementary Material

Supplementary figures.

## Abbreviations

α-SMA: α-smooth muscle actin
C6: coumarin-6
CCK-8: cell counting kit-8
Chol: cholesterol
*cryo*-SEM: *cryo*-scanning electron microscope
DAPI: 4',6-diamidino-2-phenylindole
DMSO: dimethyl sulfoxide
DOX: doxorubicin
DSC: differential scanning calorimetry
DSPE-mPEG2000: α-[6-hydroxy-6-oxido-1,12-dioxo-9-[(1-oxooctadecyl)oxy]-5,7,11-trioxa-2-aza-6-phosphanonacos-1-yl]-ω-methoxy-poly(oxy-1,2-ethanediyl)
E-Cad: E-cadherin
EMT: Epithelial-mesenchymal transition
FBS: fetal bovine serum
HSP: heat shock protein
HSPC: hydrogenated soybean phosphatidylcholine
ICG: indocyanine green
LA: low-melting-point agarose
Lipo: liposome
MSD: mean-square displacement
NIR: near-infrared
PBS: phosphate buffered saline
PDI: polymer dispersity index
PMSF: phenylmethylsulfonyl fluoride
PTT: photothermal therapy
QUE: quercetin
SVH: simulated vitreous humour
TBS-T: tris buffered saline with tween-20
TEM: transmission electron microscope
UV-Vis: ultraviolet and visible spectrophotometry
Vim: vimentin
