# Supplementary material for: Hybrid NIR-responsive liposome/hydrogel platform mediating chemo-photothermal therapy of retinoblastoma enhanced by quercetin as an adjuvant
Source: Theranostics. 2025 Mar 10;15(9):3995–4015. doi: 10.7150/thno.108471 (PMC11980669; doi:10.7150/thno.108471)
Supplement: Supplementary file 1 — Supplementary figures. [file thnov15p3995s1.pdf]

Supporting Information

Hybrid NIR-responsive liposome/hydrogel platform mediating chemo-photothermal therapy of retinoblastoma enhanced by quercetin as an adjuvant

Min Lin[1, #], Xiumei Liu[1, #], Jing Li[1], Hong Zou[2], Jie Wang[1], Zi Yan[1], Ying Liu[1], Yaqi Lyu[1, *], Nianping Feng [1, **]

[1] School of Pharmacy, Shanghai University of Traditional Chinese Medicine, Shanghai, 201203, China

[2] Department of Ophthalmology, Shuguang Hospital Affiliated to Shanghai University of Traditional Chinese Medicine, Shanghai, 201203, China.

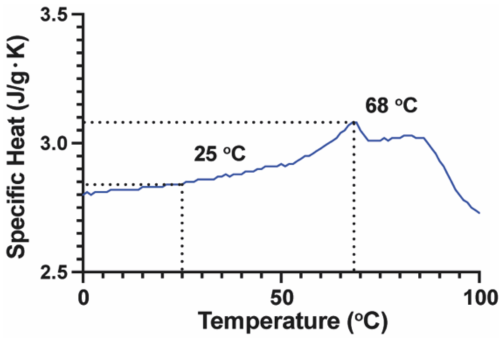

Figure S1 Heat capacity of LA measured by DSC scanning.

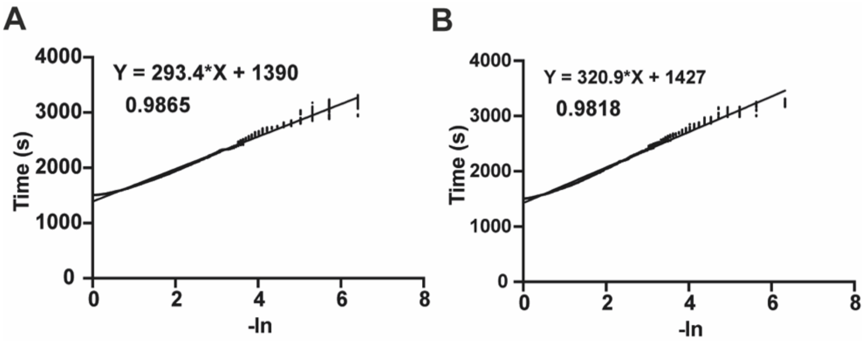

Figure S2 Linear regression of the natural logarithm of temperature change during the cooling segment. (A) After 1 cycle of "on-off" NIR irradiation. (B) After 5 cycles of "on-off" NIR irradiation. The output power of NIR irradiation was 2 W and the irradiation distance was 45 mm.

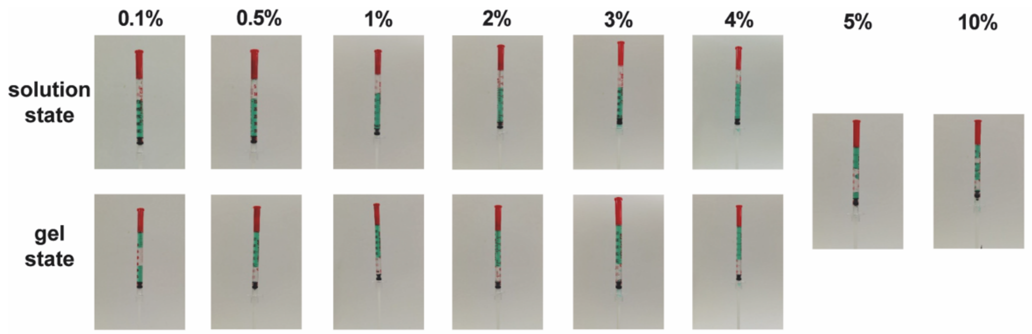

Figure S3 The appearance of LAgel in solution state or gel state at different concentrations of 0.1%, 0.5%, 1%, 2%, 3%, 4%, 5%, 10% (W/W). In the solution state, LA was dissolved in water at 70 ºC, and LA in gel state was observed at room temperature.

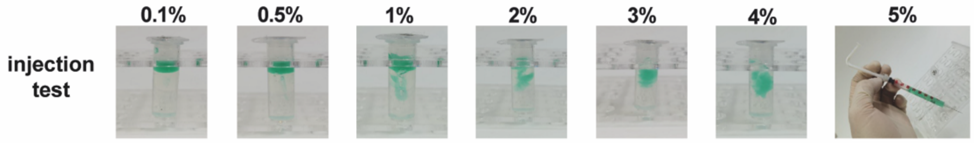

Figure S4 Injectability of LAgel at different concentrations of 0.1%, 0.5%, 1%, 2%, 3%, 4%, 5% (W/W).

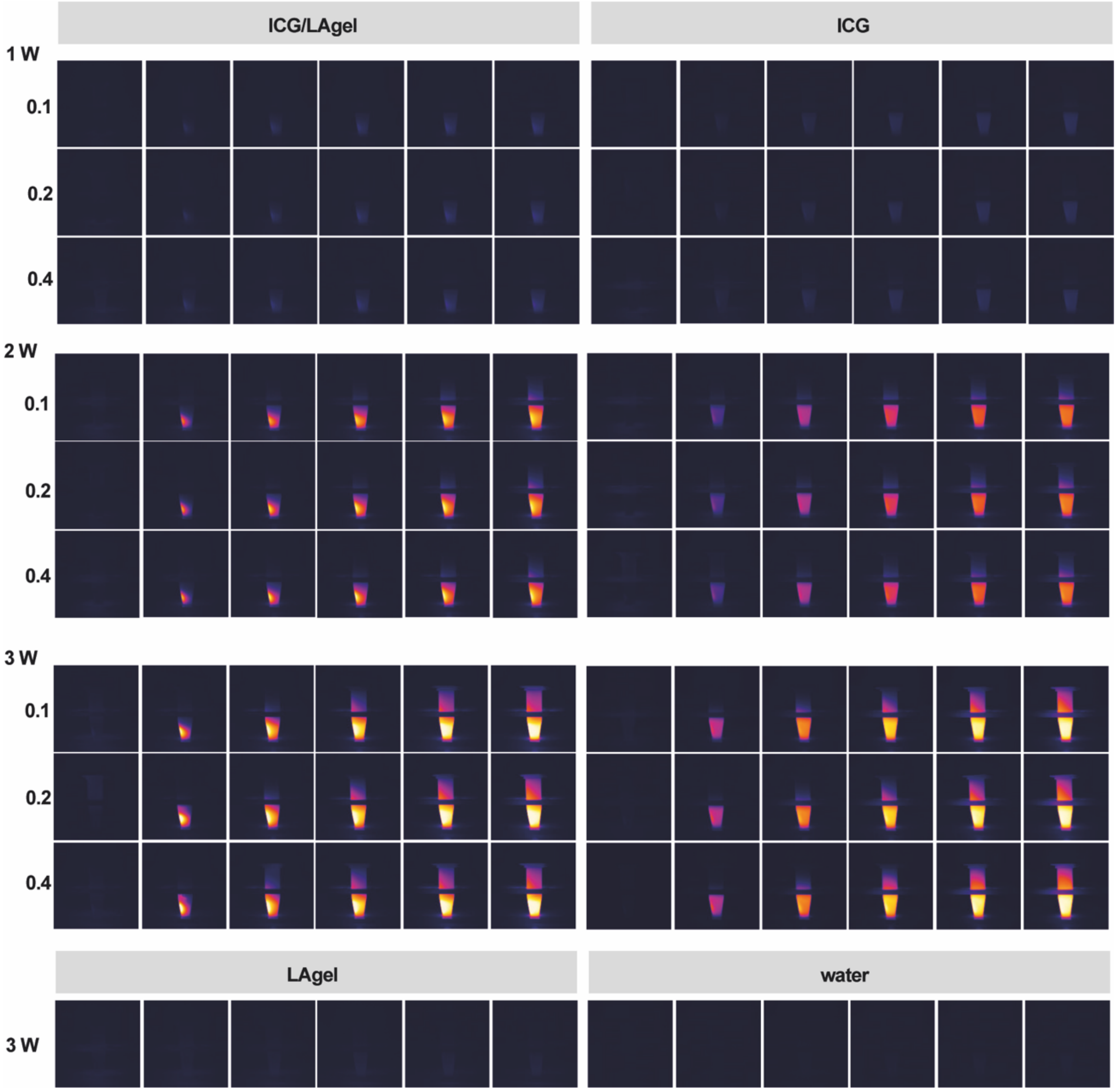

Figure S5 Thermal images of ICG/LAgel and ICG solution under NIR irradiation of 808 nm at ICG concentration of 0.1 mg/mL, 0.2 mg/mL and 0.4 mg/mL. LAgel and water were used as control. The irradiation distance was 45 mm.

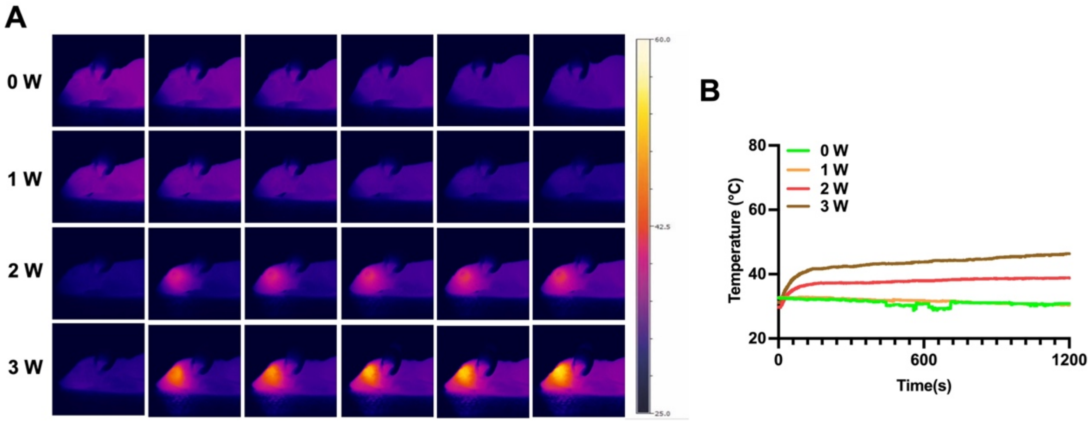

Figure S6 Thermal response of mice's eyes under sole NIR irradiation (A) Thermal photos. (B) Quantitative temperature change of the mice's eyes in Figure S6A.

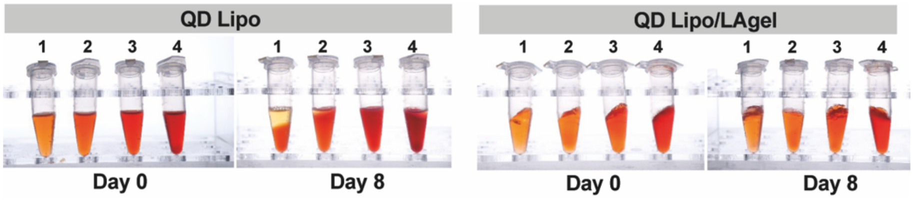

Figure S7 Stability of QD Lipo and QD Lipo/LAgel at different concentrations. The mass ratio of lipid component in QD Lipo and LAgel was (1) 0.25:1, (2) 0.5:1, (3) 1:1 and (4) 2:1.

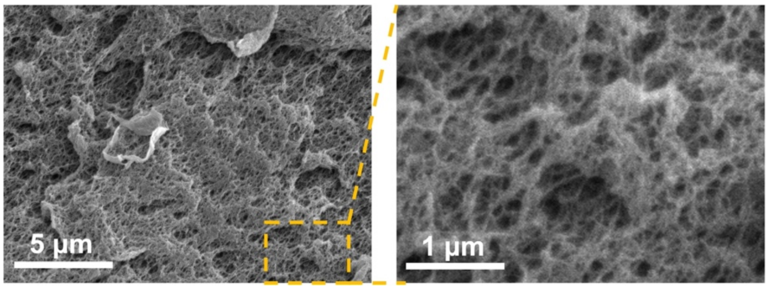

Figure S8 SEM images of LAgel.

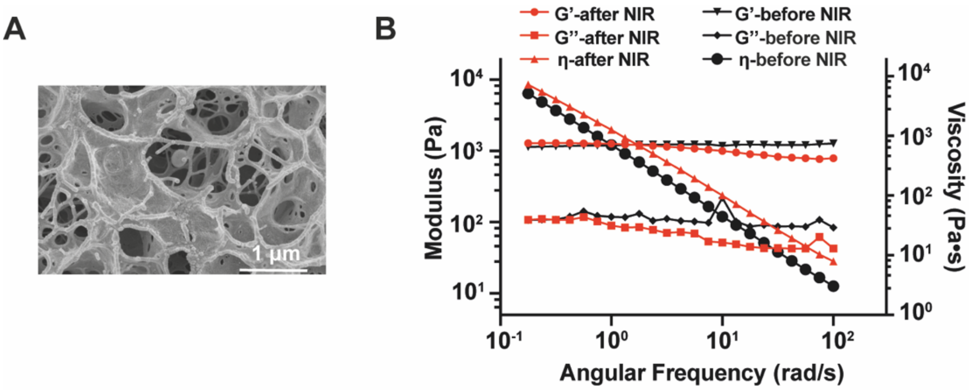

Figure S9 Characterization of QD Lipo/ICG/LAgel after NIR irradiation. (A) Cryo-SEM image (B) Frequency sweep of QD Lipo/LAgel at fixed strain of 0.1%.

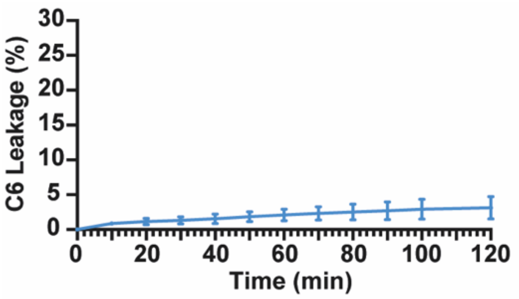

Figure S10 *In vitro* leakage profile of C6 from C6 Lipo.

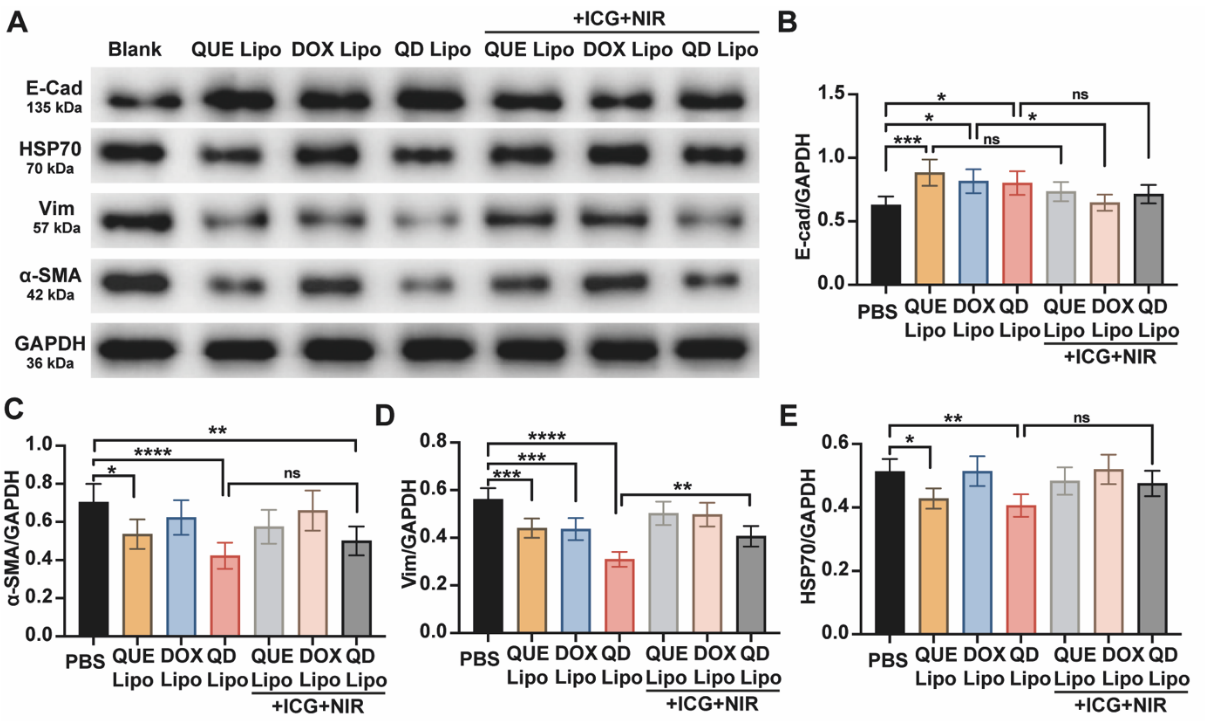

Figure S11 Inhibition of heat resistance and EMT. (A)Western blot analysis of E-cad, HSP70, α-SMA and Vim protein expression of Y79 cell lysates after treatment of PBS, QUE Lipo, DOX Lipo, QD Lipo, QUE Lipo/ICG+NIR, DOX Lipo/ICG+NIR, QD Lipo/ICG+NIR for 48 h. NIR irradiation was performed after formulation treatment for 5 min. The output power of NIR irradiation was 2 W and the irradiation distance was 45 mm. GADPH was used as a loading control. (B-E) Quantitative analysis of the expression level of the protein in Fig. S8A (means ± SD, n = 3). E-cad (B), α-SMA (C) Vim (D) and HSP70 (E). (*P < 0.05, **P < 0.01, ***P< 0.001, ****P< 0.0001)

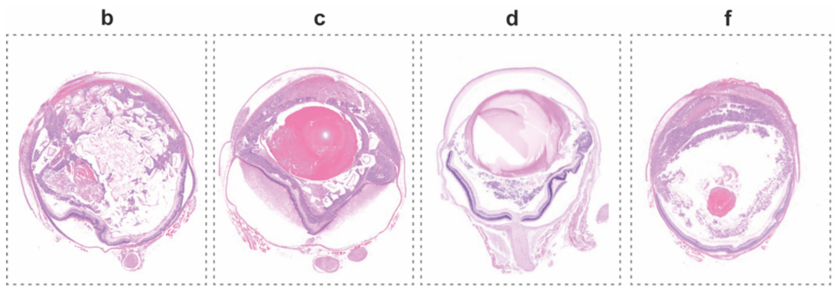

Figure S12 H&E staining of harvested eyes after treatment. The groups labelled b,c,d and f stand for the mice treated with ICG/LAgel, ICG/LAgel+NIR, QD Lipo/ICG/LAgel and QD Lipo, respectively.

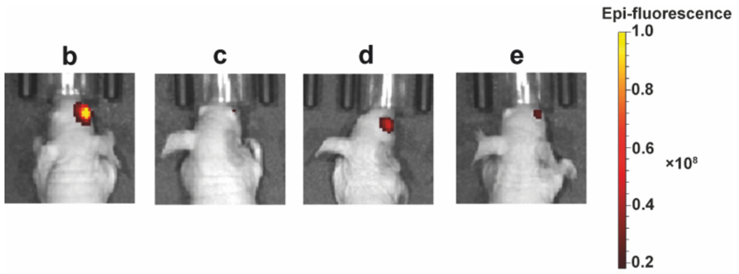

Figure S13 *In vivo* ICG fluorescence images of the eyes in Y79-GFP-luc tumour-bearing mice after treatment with ICG-containing formulations on Day 15. The groups labelled b-e stand for the mice treated with ICG/LAgel, ICG/LAgel+NIR, QD Lipo/ICG/LAgel and QD Lipo/ICG/LAgel+NIR, respectively.

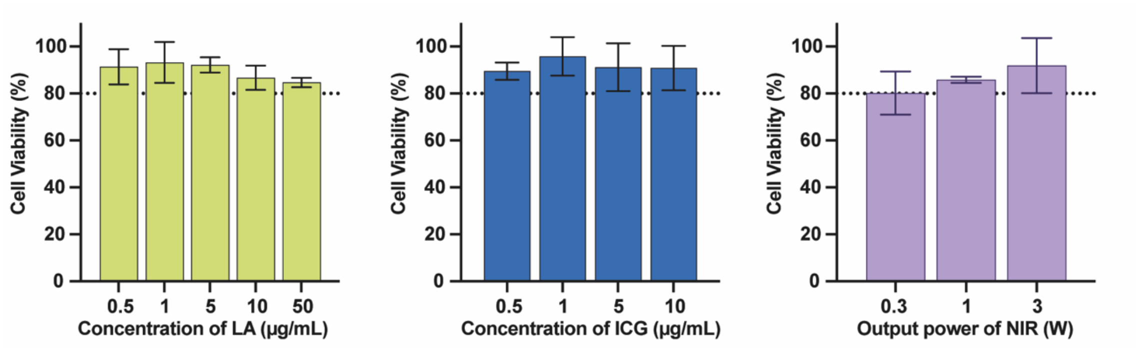

Figure S14 Cell viability of LA solution, ICG and NIR irradiation on ARPE-9 cells. (means ± SD, n = 3).

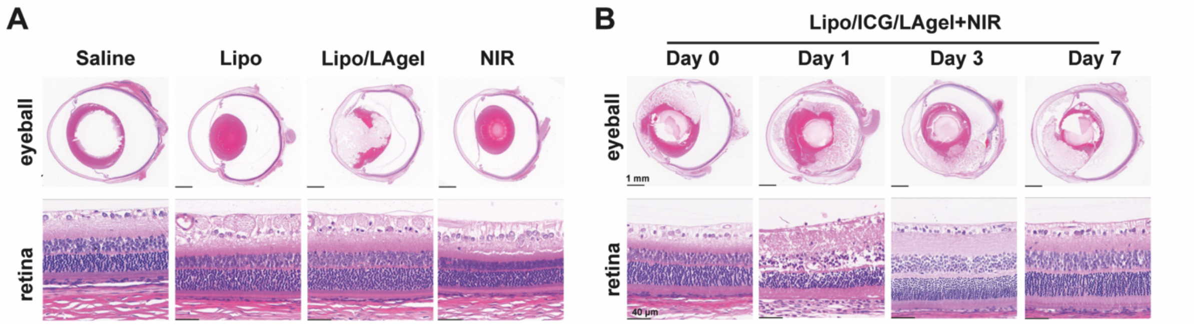

Figure S15 H&E staining of the eyeballs harvested from rats after treatment for 7 days. (A) Saline, Lipo and Lipo/LAgel were administered intravitreally. NIR irradiation of 808 nm was performed immediately after saline injection for 5 min. (B) Lipo/ICG/LAgel was intravitreally injected into the rat's eyes followed by 808 nm irradiation on Day 0, Day 1, Day 3 and Day7 after injection. The volume of preparations for intravitreal injection was 2 μL. The output power of NIR irradiation was 2 W and the irradiation distance was 45 mm.
